# Benchmarking Sparse Variable Selection Methods for Genomic Data Analyses

**DOI:** 10.1002/sim.70428

**Published:** 2026-02-10

**Authors:** Hema Sri Sai Kollipara, Tapabrata Maiti, Sanjukta Chakraborty, Samiran Sinha

**Affiliations:** ^1^ Department of Statistics & Probability Michigan State University East Lansing Michigan USA; ^2^ Department of Medical Physiology, Texas A&M Health Science Center College of Medicine Bryan Texas USA; ^3^ Department of Statistics Texas A&M University, College Station Texas USA

**Keywords:** Bayesian variable selection, false negatives, false positives, prediction, regularized regression, RNA sequence data

## Abstract

Genomics and other studies encounter many features and a selection of essential features with high accuracy is desired. In recent years, there has been a significant advancement in the use of Bayesian inference for variable (or feature) selection. However, there needs to be more practical information regarding their implementation and assessment of their relative performance. Our goal in this paper is to perform a comparative analysis of approaches, mainly from different Bayesian genres that apply to genomic analysis. In particular, we are examining how well shrinkage, global–local, and mixture priors, SUSIE, and a simple two‐step procedure—namely, RFSFS, which we propose—perform in terms of several metrics: FDR, FNR, *F*‐score, and mean squared prediction error under various simulation scenarios. There is no single method that outperforms others uniformly across all scenarios and in terms of variable selection and prediction performance metrics. So, we order the methods based on the average ranking across different scenarios. We found LASSO, spike‐and‐slab prior with normal slab (SN), and RFSFS are the most competitive methods for FDR and *F*‐score when features are uncorrelated. When features are correlated, SN, SuSIE, and RFSFS are the most competitive methods for FDR whereas LASSO has an edge over SuSIE in terms of *F*‐score. For illustration, we have applied these methods to analyzed The Cancer Genome Atlas Program (TCGA) renal cell carcinoma (RCC) data and have offered methodological direction.

## 1 Introduction

Feature selection is ubiquitous in many disciplines, specially in genomic studies. In genomic studies, thousands of transcriptomics, such as RNA sequence, protein expressions, mutations, methylations, are measured for every subject or experimental unit. Due to the cost the number of the experimental units is much smaller than the dimension of the transcriptomics. It is believed that only a small percentage of thousands of transcriptomics is important for regulating a phenotype. Identification or selection of these important features is thus the primary task, which help develop strategies to control the phenotype.

In this paper, we aim to compare different feature selection methods in a multiple linear *mean* regression setup, where the phenotype or the outcome of interest is numeric. Here features are the explanatory variables. One of the key aspects is the length (dimension) of the feature is much larger than the sample size (i.e., the number of parameters p≫n), so the least‐square method cannot be applied for estimating the regression parameters. To circumvent the situation, the regularized regression method is used, where the regression parameters are estimated by minimizing the error sum of squares under the sparsity constraint, which results in a solution with many components of the regression parameter vector to be zero. A sparse solution is usually obtained by penalizing the L1 norm of the regression parameter. Although Lp norm with 0<p<1 also yields a sparse solution, it is not so popular due to the non‐convex optimization.

Analogous to the regularized regression, in the Bayesian method, constrains are imposed via the prior distribution for the parameters. Our simulation‐based comparison includes Bayesian approaches for variable selection in a multiple linear regression setup, which encompass three kinds of priors, (1) the shrinkage prior, such as Bayesian LASSO [1], and (2) global–local shrinkage priors, such as Horseshoe [2], Horseshoe+ [3] and regularized Horseshoe [4] and (3) the spike‐and‐slab priors, also referred to as mixture priors, consist of a spike component typically a point mass at zero and a slab component representing the distribution of nonzero effects, such as a normal distribution [5, 6] or a double‐exponential (Laplace) distribution. Additionally, we include the recently developed method, called the sum of single effects linear regression (SUSIE) [7] in the comparison. While we discuss the details of these methods in the later section, we now briefly talk about the existing literature that numerically compared different variable selection techniques and the necessity of this benchmarking.

O'hara et al. [8] offered a comprehensive overview of Bayesian approaches to variable selection that encompassed the Gibbs variable selection method of [9], the stochastic search variable selection (SSVS) method of [10], the adaptive shrinkage with Jeffreys' or Laplacian priors, and the reversible jump MCMC from the perspective of sparsity, adaptation, integration, and the Bayesian model averaging. The authors systematically reviewed the methods based on model formulation, computational strategies, and theoretical underpinnings, all with p much smaller than n. Bhadra et al. [11] comprehensively explored the intersection between two prominent statistical techniques, LASSO and horseshoe regularization. The survey delved into these theoretical foundations, computational aspects, and methodological developments associated with these high‐dimensional estimation methods. They compared LASSO, Horseshoe, Horseshoe+, Dirichlet‐Laplace prior through numerical studies for the p<n case. Lu and Lou [12] conducted a comparative study of LASSO, Ridge, elastic‐net, group LASSO, adaptive LASSO, fused LASSO, and stepwise variable selection techniques with different levels of sparsity (10% and 30%), and different choices of (n,p) for the n>p case.

On the large p small n context, Piironen et al. [4] reviewed horseshoe priors and addressed challenges associated with (1) specifying a prior for the global shrinkage hyperparameter based on the sparsity of the parameter vector, and (2) how to separately specify information about sparsity and the amount of regularization for the largest coefficients led them to consider a regularized horseshoe prior. The authors numerically compared the regularized horseshoe with the spike‐and‐slab prior with a normal distribution for the slab part for the p≥n case. Malsiner‐Walli et al. [13] thoroughly examined different spike‐and‐slab priors within the context of Bayesian variable selection. The paper systematically compared the performance of various Markov Chain Monte Carlo (MCMC) implementations for different spike‐and‐slab priors with multiple choices of the slab part, emphasizing the assessment of posterior inclusion probabilities and sampling efficiency using simulated data for the p≈n case.

While global–local shrinkage priors and spike‐and‐slab priors are widely used for estimating high‐dimensional regression parameters, mostly in a sparse situation, variable selection is still a challenging task. Li and Pati [14] proposed a variable selection technique employing shrinkage priors to tackle these challenges with a minimal number of tuning parameters, and the authors claimed that the method performed better in the presence of high collinearity in covariates. Recently, Wang et al. [7] proposed a novel technique for variables selection in regression models. This method is especially useful in scenarios where there is a high degree of correlation among the covariates. In certain applications, the selection of one of the highly correlated variables arbitrarily may be sufficient for building an accurate predictor. It aims to provide a user‐friendly and effective approach to variable selection in regression analyses, particularly in the domain of genetic studies.

More recently, Fan et al. [15] provided a review and small‐scale benchmarking study with six methods including Bayesian LASSO and the spike‐and‐slab prior. They emphasized on credible interval coverage and uncertainty quantification in high‐dimensional Bayesian models, particularly under robust formulations. Their study, however, is limited to a subset of robust Bayesian methods and a few frequentist comparators, and does not provide a broad evaluation of selection accuracy, predictive performance, or computational efficiency. Our work complements theirs by systematically comparing Bayesian and frequentist methods across a wider range of priors, metrics, and simulation scenarios.

Besides all these important works, the literature lacks a sweeping comparison of methods from different Bayesian genres pertinent to genomic analysis. To the best of our knowledge, this is the first study to systematically compare Bayesian (a) shrinkage, (b) global–local, and (c) mixture priors, (d) SUSIE, and (e) the classical approaches, LASSO, elastic‐net, adaptive LASSO and our newly proposed two‐step procedure, RFSFS (Random Forest Screening followed by Forward Selection), which integrates random forest and stepwise regression to enhance variable selection. Such comparison will enrich the feature selection literature and guide practitioners in choosing the best available method following their needs. Among other novelties, (1) we are focusing on the p≫n case with (a) different sparsity levels and (b) different signal‐to‐noise ratios (SNR), focusing on the structure of the genomic data; (2) we are employing different performance metrics, including the false discovery rate (FDR), false negative rate (FNR), *F*‐score, and computational time; and (3) finally, we apply these methods to analyze the kidney (renal) clear cell carcinoma (RCC) data [16] obtained from the TCGA hub to identify important miRNAs regulating the proto‐oncogene MET.

The analyses of the data by thirteen different methods provide exploratory evidence for the identified miRNAs rather than confirmatory claims. Separate analyses for the male and female groups, and the identification of distinct sets of miRNAs, highlight potential gender‐related differences in the molecular etiology of RCC. Our work is different from identifying important miRNAs using diverse classification techniques like support vector machines, artificial neural networks, decision trees, random forests, and naive Bayes, that correlate with cancer progression or cancer subtypes. Instead, we are seeking to identify important miRNAs that interact with a proto‐oncogene. This approach aims to uncover the regulatory roles of miRNAs in oncogenesis by examining their influence on genes that play crucial roles in cancer development and progression [17]. While recent studies such as [15] have emphasized inference quality in high‐dimensional models, our emphasis is on feature selection and prediction, with post‐selection inference acknowledged as an open challenge and important direction for future work.

## 2 Background

Suppose that we have data from n subjects and use index i to denote a subject, so i=1,…,n. For the *i*th subject, let $Y_{i}$ be the outcome and $X_{i}={\left(X_{i,1},\ldots ,X_{i,p}\right)}^{⊤}$ be the p features. We use D to denote the observed data, that means $D=\left\{X_{i},Y_{i},i=1,\ldots ,n\right\}$. For our motivating example, Y denotes the expression of a gene and X denotes the expression of several miRNAs. Without loss of generality, we assume that the conditional mean of Y is zero when each feature is centered. The regression model is

$$Y_{i}=\sum_{j=1}^{p}X_{i,j}\beta_{j}+\varepsilon_{i},$$

where the random noise $\varepsilon_{i}$ are independent and identically distributed as $\text{Normal}\;\left(0,\sigma^{2}\right)$, and are independent of $X_{i}$. The model unknown parameters are $\beta ={\left(\beta_{1},\ldots ,\beta_{p}\right)}^{⊤}$ and $\sigma^{2}$. We focus on the sparse situation where p≫n and most of the β parameters are zero. Our goals are (1) identifying non‐zero regression parameters and (2) optimally estimating the non‐zero β parameters. Identifying non‐zero β's is equivalent to identifying features that exert non‐zero effect on the outcome.

For reducing the over‐fitting problem in the presence of a large number of parameters, in the classical inference, parameters are estimated by maximizing a penalized log‐likelihood function that is equivalent to

(1) $$\hat{\beta }=\arg\min_{\beta }\;\sum_{i=1}^{n}{\left(Y_{i}-X_{i}^{⊤}\beta \right)}^{2}+{\mathrm{pen}}_{\zeta }\left(\beta \right),$$

where ${\mathrm{pen}}_{\zeta }$ is the penalty term. If ${\mathrm{pen}}_{\zeta }\left(\beta \right)$ is the negative of the logarithm of a prior density, then $\hat{\beta }$ can be interpreted as the posterior mode of the β. The non‐negative parameter ζ is called the shrinkage parameter. For different choices of ${\mathrm{pen}}_{\zeta }\left(\beta \right)$ we obtain several well‐known regularized estimators such as Ridge, LASSO, SCAD, Adaptive LASSO, etc.

## 3 Methods To Be Compared

### 3.1 Classical Regularized Regression: LASSO, Elastic Net

Among different regularization techniques, the Least Absolute Shrinkage and Selection Operation (LASSO) method, also known as $\ell_{1}$ penalty, is widely used for variable selection. The penalty is $p{\mathrm{en}}_{\zeta }\left(\beta \right)=\zeta \sum_{j}|\beta_{j}|$. LASSO tends to select only one variable from a group of highly correlated variables due to no grouping property, and its performance seems to be unstable in the presence of multicollinearity [18, 19, 20]. To overcome this challenge, one can employ the elastic‐net method which carries a mixture of $\ell_{1}$ (LASSO) and $\ell_{2}$ (ridge) penalty, $p{\mathrm{en}}_{\zeta }\left(\beta \right)=\sum_{j}\left(\zeta_{1}|\beta_{j}|+\zeta_{2}\beta_{j}^{2}\right)$.

Elastic‐net [21] (1) tends to perform better than LASSO in predicting and selecting correlated features, and (2) has no limitation on the number of selected features. These all are convex optimization, which is computationally much simpler than the non‐convex optimization for $\ell_{q}$ penalty with q∈(0,1). Although selection and estimation are natural outcomes of regularized methods, the inference of the regression parameters of the selected features is made separately, called post‐selection inference [22].

The regularized estimators also admit a Bayesian interpretation: they can be viewed as posterior modes under specific prior distributions. For example, the LASSO estimator can be seen as the posterior mode under independent double‐exponential (Laplace) priors on the coefficients.

Park and Casella [1] introduced the Bayesian LASSO by assigning $\pi \left(\beta_{j}|\lambda ,\sigma^{2}\right)\propto \lambda /\left(2\sqrt{\sigma^{2}}\right)\exp\;\left(-\lambda |\beta_{j}|/\sqrt{\sigma^{2}}\right),$ together with $\pi \left(\sigma^{2}\right)\propto \sigma^{-2}$ and a Gamma prior on $\lambda^{2}$. This hierarchical formulation allows the shrinkage parameter to be learned from the data, unlike the classical LASSO where it is fixed. The Bayesian LASSO coincides with the classical LASSO at the posterior mode but further enables full posterior inference, thereby providing credible intervals and uncertainty quantification.

Elastic net also admits a Bayesian interpretation. Specifically, its penalty corresponds to a prior that is a scale mixture of normals, where the $\ell_{1}$ part induces sparsity and the $\ell_{2}$ part stabilizes estimation in the presence of multicollinearity. Hans [23] showed that the elastic net estimator can be interpreted as the posterior mode under such a mixture prior, where the scale parameter follows a truncated inverse‐gamma distribution.

### 3.2 Adaptive LASSO

The adaptive LASSO (ALASSO) [24] extends the classical LASSO by introducing variable‐specific weights in the $\ell_{1}$ penalty, thereby reducing shrinkage for strong signals and increasing shrinkage for weaker ones. Formally, the estimator is defined as

$$\hat{\beta }=\arg\min_{\beta }\;\sum_{i=1}^{n}{\left(Y_{i}-\sum_{j=1}^{p}\beta_{j}X_{\mathrm{ij}}\right)}^{2}+\lambda \sum_{j=1}^{p}\;\omega_{j}|\beta_{j}|,$$

where for data‐adaptive weights, one can set $\omega_{j}=1/|{\hat{\beta }}_{j}^{\text{init}}|$ with ${\hat{\beta }}_{j}^{i\mathrm{nit}}$ being the initial estimate of the $j^{\mathrm{th}}$ regression coefficient (e.g., the ridge estimator).

Compared with LASSO, the adaptive LASSO enjoys the oracle property under mild conditions: it can correctly identify the true model with probability tending to one and produce asymptotically unbiased estimates for the nonzero coefficients. Tuning of the regularization parameter λ is typically performed via cross‐validation.

### 3.3 Horseshoe Priors: Horseshoe, Horseshoe+, Regularized Horseshoe

Horseshoe is a hierarchical Bayesian model having a continuous shrinkage prior [2], and it is

(2) $$\begin{array}{ll} \beta_{j}|\lambda_{j},\tau & ∼\text{Normal}\left(0,\tau^{2}\lambda_{j}^{2}\right),\lambda_{j}∼C^{+}\left(0,1\right),\;j=1,\cdots ,p,\\ \;\tau & ∼C^{+}\left(0,1\right), \end{array}$$

where $C^{+}\left(0,1\right)$ denotes the half‐Cauchy distribution. Parameter $\lambda_{j}$'s are referred to as the local shrinkage parameters and τ as the global shrinkage parameter. The prior distribution for $\sigma^{2}$ is assumed to be proportional to $\sigma^{-2}$, that is, $\pi \left(\sigma^{2}\right)\propto \sigma^{-2}$ (Jeffrey's prior). Each $\beta_{j}$ is assumed to be conditionally independent. The horseshoe prior is useful due to its two interesting properties: (1) it allows strong signals to remain large due to flat Cauchy‐like tails, and (2) it allows severe shrinkage due to an infinitely tall spike at the origin. The idea is that posterior distribution of the β‐parameters that are truly zero will likely be concentrated around zero, while the posterior of nonzero β's will mostly be around a nonzero value.

The horseshoe prior does not allow passing information on sparsity and the amount of regularization for the largest coefficients, separately. This makes the horseshoe prior not useful for weakly identified models or data with separations. Furthermore, the horseshoe prior may encounter difficulties in accurately estimating signals characterized by large amplitudes, leading to decreased precision and slower convergence. Technically, its inadaptability to different sparsity patterns can impede its effectiveness in capturing the true underlying structure of sparse signals. To address some of these challenges, Bhadra et al. and Makalic et al. [3, 25] proposed the horseshoe+ prior

(3) $$\begin{array}{ll} \beta_{j}|\lambda_{j},\tau & ∼\text{Normal}\left(0,\tau^{2}\lambda_{j}^{2}\right),\lambda_{j}|\eta_{j}∼C^{+}\left(0,\eta_{j}\right),\eta_{j}∼C^{+}\left(0,1\right),\\ & \;j=1,\cdots ,p,\\ \;\tau & ∼C^{+}\left(0,1\right), \end{array}$$

and $\pi \left(\sigma^{2}\right)\propto \sigma^{-2}$. The scale $\eta_{j}$ dynamically changes the shape of the prior of the shrinkage term across the features, as opposed to the same shrinkage prior for every feature.

Due to the Cauchy‐like tails for the priors for the local and global scale parameters of horseshoe and horseshoe+, some regression coefficients could be too large. To address this concern, Piironen et al. [4] proposed the regularized Horseshoe prior

$$\beta_{j}|\lambda_{j},\tau ,c∼\text{Normal}\left(0,\tau^{2}{\tilde{\lambda }}_{j}^{2}\right),\;{\tilde{\lambda }}_{j}^{2}=\frac{c^{2}\lambda_{j}^{2}}{c^{2}+\tau^{2}\lambda_{j}^{2}},\;\lambda_{j}∼C^{+}\left(0,1\right),$$

where c>0 and the authors used an inverse‐gamma prior on $c^{2}$. The authors used different priors for τ, one is $C^{+}\left(0,1\right)$ and the other is $C^{+}\left(0,\tau_{0}^{2}\right)$ with $\tau_{0}^{2}$ being fixed to $p_{0}/\left(n-p_{0}\right)$. This prior guarantees shrinking of all coefficients at least by a small amount. The intuition is when $\tau^{2}\to 0$ and $\tau^{2}\lambda_{j}^{2}\ll c^{2}$, then ${\tilde{\lambda }}_{j}^{2}\to \lambda_{j}^{2}$ and $\beta_{j}\to 0$, on the other hand when $\tau^{2}\lambda_{j}^{2}\gg c^{2},{\tilde{\lambda }}_{j}^{2}\to c^{2}/\tau^{2}$, and consequently $\beta_{j}$ follows $\text{normal}\left(0,c^{2}\right)$. Like horseshoe, this prior shrinks the small signals to zero but regularizes even the largest coefficients. As we know, controlling the amount of shrinkage for the largest coefficients would be useful for reducing the mean squared error for the regression parameters and, especially when there is some separation issue. Of course, separation does not arise for a numeric outcome as in our case. Consistent with our evaluation protocol, these continuous‐shrinkage priors do not produce exact zeros; we therefore apply a data‐adaptive thresholding via clustering at the analysis stage to obtain a binary selection (see Section 4.3). Just note that none of these horseshoe methods is designed to address correlated features [14].

### 3.4 Spike‐and‐Slab Priors

The spike‐and‐slab prior is a popular Bayesian tool for inducing sparsity in high‐dimensional regression problems. It is typically formulated as a mixture model where each regression coefficient is drawn from either a “spike” distribution, which heavily shrinks the coefficient toward zero, or a more diffuse “slab” distribution that allows greater flexibility. This can be expressed hierarchically as:

(4) $$\begin{array}{c} \beta_{j}|\lambda_{j}\overset{\text{indep}}{∼}\left(1-\lambda_{j}\right)\psi_{0}\left(\beta_{j}\right)+\lambda_{j}\psi_{1}\left(\beta_{j}\right),\lambda_{j}\overset{\text{indep}}{∼}\text{Bernoulli}\left(\omega_{j}\right)\\ \;\text{for}\;j=1,\ldots ,p. \end{array}$$

Without any prior information, $\omega_{j}$'s are set to one‐half, which is called an indifference prior. Here $\psi_{0}\left(\beta_{j}\right)$ and $\psi_{1}\left(\beta_{j}\right)$ represent the spike and slab distributions, respectively. When both components are normal distributions with small (ε) and large (c) variances, respectively, this yields the classical spike‐and‐slab model, often referred to as the stochastic search variable selection (SSVS) approach. We refer to this implementation as the SN (the spike‐and‐slab prior with the normal slab) model. It introduces binary inclusion indicators to explicitly control whether each coefficient is active (nonzero) or inactive (zero), and allows the inclusion probabilities to be governed by a Beta hyperprior. Posterior inclusion probabilities $\mathrm{Pr}\left(\lambda_{j}=1|\text{data}\right)$ provide a direct measure of variable relevance, making the SN prior attractive for uncertainty quantification in variable selection. The Gaussian slab ensures coefficients remain finite while the spike enforces strong shrinkage toward zero, yielding sparse solutions. This formulation corresponds to the SSVS framework of [10, 26] and has been widely adopted in high‐dimensional regression due to its interpretability and theoretical grounding. Although c≫ε, too small ε may cause MCMC sampling to be stuck at a local value [26]. In SSVS, an inverse‐gamma prior is used for $\sigma^{2}$, and ω is generally set to the sparsity level.

Note that the spike‐and‐slab prior with the slab being normal and spike part being a degenerate distribution at zero can be expressed as $\beta_{j}|\lambda_{j},c∼N\left(0,c^{2}\lambda_{j}^{2}\right),\lambda_{j}∼\text{Bernoulli}\left(\pi \right),$ with π being a scalar, which has a resemblance with the regularized horseshoe prior with unit global scale parameter and c<∞. Since continuous shrinkage does not produce exact zeros, binary classification of variables under SN is achieved by thresholding posterior inclusion probabilities.

An alternative formulation, known as the spike‐and‐slab Lasso (SSLASSO), avoids discrete binary indicators by assigning each coefficient a Laplace prior with a scale parameter drawn from a two‐component exponential mixture. Specifically, each $\beta_{j}$ is modeled as:

(5) $$\beta_{j}∼\text{Laplace}\left(0,\lambda_{j}\right),\;\lambda_{j}^{2}∼\left(1-\pi \right)\cdot \mathrm{Exp}\left(\zeta_{0}^{2}/2\right)+\pi \cdot \mathrm{Exp}\left(\zeta_{1}^{2}/2\right).$$

This prior, introduced by Ročková et al. [27], induces a non‐separable, self‐adaptive penalty that imposes strong shrinkage (through smaller scale $\zeta_{0}$) on small coefficients and weak shrinkage (through larger $\zeta_{1}$) on large coefficients. This adaptivity allows SSLASSO to correct for multiplicity and reduce bias in variable selection, offering behavior similar to classical spike‐and‐slab models but without relying on latent binary inclusion indicators. Notably, SSLASSO reduces to the classical LASSO when the spike and slab shrinkage levels are identical (i.e., $\zeta_{0}=\zeta_{1}$), resulting in a uniform Laplace prior and eliminating the adaptive shrinkage behavior. Unlike the formulation in Equation (4), the mixture structure in SSLASSO applies to the prior scale, resulting in a continuous shrinkage prior that is computationally efficient and well‐suited for high‐dimensional regression.

### 3.5 Sum of Single Effects Regression Model

The Sum of Single Effects regression model (SuSiE) is a new approach that quantifies the uncertainty of selecting a feature [7]. The idea is to write the vector of regression parameters β as a sum of L vectors, where each vector is termed as a “single effect” vector. Each single effect vector comprises exactly one nonzero element. This representation allows β to be expressed in terms of at most L nonzero elements, where L, a user‐specified parameter, denotes the maximum number of effects. The β‐vector is written as

(6) $$\begin{array}{ll} \beta & =\sum_{l=1}^{L}b_{l},b_{l}={\left(b_{l,1},\ldots ,b_{l,p}\right)}^{⊤}\in {ℛ}^{p},\\ b_{l} & =\gamma_{l}g_{l},\gamma_{l}={\left(\gamma_{l,1},\ldots ,\gamma_{l,p}\right)}^{⊤}\in {\left\{0,1\right\}}^{p},g_{l}\in ℛ,\\ \gamma_{l} & ∼\text{Mult}\left(1,\pi_{l}\right),\;\pi_{l}={\left(\pi_{l,1},\ldots ,\pi_{l,p}\right)}^{⊤}\in {\left(0,1\right)}^{p},\pi_{l,1}+\cdots +\pi_{l,p}=1,\\ g_{l} & ∼\text{Normal}\left(0,\tau_{l}^{2}\right), \end{array}$$

Typically, the residual variance $\sigma^{2}$, and the hyperparameters $\tau^{2}={\left(\tau_{1}^{2},\ldots ,\tau_{L}^{2}\right)}^{⊤}$ and $\pi_{1},\ldots ,\pi_{L}$ are estimated or can be fixed at desired values. The authors used min(10,p) as the default value of L in the absence of any prior values of L. SuSiE is a generalization of the single effect regression (SER) where only one feature has a nonzero effect (i.e., L=1). If L≪p, then SuSiE is approximately equal to the Bayesian variable selection regression (BVSR) method of [6]. SuSiE's distinctive structure facilitates a streamlined and intuitive model fitting procedure known as Iterative Bayesian Stepwise Selection (IBSS), providing a Bayesian analog of traditional stepwise selection methods with significant advantages in terms of computation and when multiple features correlated compete for selection.

The authors' newly developed technique numerically obtains the posterior distribution, called iterative Bayesian Stepwise Selection (IBSS) [7]. The effect of the *j*th feature, $\beta_{j}$, is estimated by the posterior mean of $b^{\left(j\right)}=\sum_{l=1}^{L}\;b_{l,j}$ which is zero if and only if $b_{l,j}=\gamma_{l,j}g_{l}=0$ for all l.

### 3.6 SIS+LASSO (Sure Independence Screening With LASSO)

Sure Independence Screening (SIS) was proposed by Fan and Lv [28] to address ultrahigh‐dimensional regression problems where p≫n. The central idea is to reduce dimensionality by ranking predictors according to their marginal correlation with the response.

Let $\omega_{j}$ be the correlation between the outcome Y and the $j^{\mathrm{th}}$ predictor. Predictors are then ranked by $|\omega_{j}|$, and the top

$$d_{n}=\lfloor n/\log n\rfloor$$

variables are retained to form the screening set $S_{\mathrm{SIS}}$. In the second stage, the LASSO estimator is obtained on the reduced feature set. Specifically,

$$\hat{\beta }\left(\lambda \right)\in \arg\min_{\beta \in {\mathbb{R}}^{d_{n}}}\left\{\frac{1}{2n}∥Y-X_{S_{\mathrm{SIS}}}\beta {∥}_{2}^{2}+\lambda ∥\beta {∥}_{1}\right\},$$

where the tuning parameter λ is selected by 10–fold cross‐validation. The final selected model consists of the nonzero coefficients in $\hat{\beta }\left(\lambda \right)$.

This hybrid procedure combines the computational efficiency of SIS with the adaptivity of LASSO. By restricting the feature space to O(n) predictors, SIS+LASSO mitigates the instability of direct LASSO in ultrahigh dimensions and improves scalability. However, SIS relies on marginal correlations and may fail to capture predictors with weak marginal but strong joint effects, especially under high collinearity. Despite these limitations, SIS+LASSO is a widely used two‐stage method that provides a useful benchmark in high‐dimensional feature selection.

### 3.7 RF (Random Forest)

Random forests [29] are ensemble learning methods that construct multiple decision trees using bootstrap samples of the training data. At each split within a tree, a random subset of features is considered, introducing randomness that decorrelates the trees. The final prediction is obtained by aggregating the outputs of all trees—via majority vote for classification or averaging for regression. Features were ranked using the minimum‐depth criterion, which prioritizes variables that tend to split closer to the tree root, and the top $d_{n}=\lfloor n/\log n\rfloor$ predictors were retained for subsequent selection. This ensemble strategy reduces variance while maintaining low bias, making random forests robust to overfitting and effective for high‐dimensional and nonlinear problems.

Alternative random forest–based feature selection approaches, such as conditional inference forests and permutation importance measures [30], are tend to be computationally intensive and less transparent, and can be difficult to interpret.

### 3.8 RFSFS (Random Forest Screening Followed by Forward Selection With BIC)

We propose a two‐step variable selection procedure, called RFSFS, that integrates random forest screening [29, 31] with stepwise forward selection [32, 33, 34] under the Bayesian Information Criterion (BIC). To our knowledge, this specific integration of minimum depth screening with BIC‐guided forward selection has not been systematically examined in previous comparative studies.

In the first stage, predictors are ranked using the *minimum depth* criterion from random forest [35]. For each feature j, the average depth at which it first appears in a tree is computed, and variables with smaller average depth are considered more important. Like [28], only the top ⌊n/log(n)⌋ predictors are kept but based on the smallest minimum depth values, yielding the screening set

$$\begin{array}{cc} S_{\mathrm{RF}} & =\left\{j:\mathrm{rank}\left(\;\text{average}\;\text{depth}\;\text{of}\;\text{the}\;j^{\mathrm{th}}\;\text{feature}\;\right)\right.\\ & \;\left.\leq \lfloor n/\log\left(n\right)\rfloor \right\}. \end{array}$$

This step reduces dimensionality while preserving potentially informative variables. Compared with the correlation based feature selection, RF based feature selection can identify features that are non‐linearly associated with the outcome.

The second stage applies forward stepwise regression to the features $S_{\mathrm{RF}}$, with BIC guiding inclusion and elimination of the variables. Relative to SIS+LASSO, our numerical experiment (results not presented in the paper) showed that SIS combined with forward selection yields better selection accuracy and prediction error, which motivated us using the forward selection with the minimum BIC criteria at the second stage. The random forest with the minimum depth criterion is empirically effective but lacks a formal selection‐consistency proof, while forward selection with BIC inherits only partial consistency guarantees. BIC is consistent under exhaustive search [36], and greedy stepwise regression can achieve consistency only under additional conditions such as strong signals and weak correlations [37]. Despite these limitations, the combined procedure demonstrates strong empirical performance across diverse scenarios as seen in the simulation results.

## 4 Simulation Study

### 4.1 Simulation Design

#### 4.1.1 Data‐Generating Framework

Data were generated under diverse data‐generating mechanisms. The study assessed relative performance, strengths, and limitations of representative frequentist, Bayesian, and hybrid approaches across varying sparsity, signal strength, and structural complexity. The first data simulation model was $Y_{i}=X_{i,1}\beta_{1}+X_{i,2}\beta_{2}+\cdots +X_{i,p}\beta_{p}+\varepsilon_{i}$, where $X_{i}={\left(X_{i,1},\ldots ,X_{i,p}\right)}^{⊤}$ denotes the p‐dimensional predictor vector for observation i, and $\varepsilon_{i}$ represents the random error term. Unless otherwise specified, the sample size was fixed at n=200 and the number of predictors at p=1000, representing a high‐dimensional regime (p≫n). Two error distributions were considered: Gaussian noise, $\varepsilon_{i}∼\text{Normal}\left(0,\sigma^{2}\right)$, and heavy‐tailed noise, $\varepsilon_{i}∼t_{2}$, with the latter scaled to match the signal‐to‐noise ratio (SNR) of the Gaussian case. The inclusion of a heavy‐tailed error setting reflects the motivation of recent robust Bayesian frameworks, such as those proposed by [38], which emphasize model resilience under deviations from normality. The noise variance $\sigma^{2}$ was calibrated such that $\mathrm{SNR}=\mathrm{Var}\left(X\beta \right)/\sigma^{2}$ attained values of 1 and 5, representing low‐ and high‐signal environments.

Unless otherwise noted, predictors were generated independently from either Normal(0,1) for continuous covariates or Bernoulli(0.7) for binary covariates, allowing performance evaluation under both continuous and discrete feature distributions. The sparsity level, defined as the proportion s of active (nonzero) regression coefficients, was varied across {1%,3%,10%,30%} to span extreme to moderate sparsity settings. Within the active set, nonzero coefficients were randomly assigned equal numbers of positive and negative effects of comparable magnitude, producing a realistic mixture of reinforcing and opposing signals. This framework enabled systematic assessment of how each method responds to variations in noise, predictor dimensionality, and signal sparsity.

#### 4.1.2 Sparsity and Signal Regimes

Three coefficient configurations were examined: *Scenario* 0 (*Low‐Noise Benchmark*), *Scenario* 1 (*Extreme Sparsity*), and *Scenario* 2 (*Moderate Sparsity*). Scenario 0 served as a baseline with error variance fixed at σ=1 rather than calibrated to a target SNR, producing a low‐noise, high‐signal environment that provides an upper bound for achievable selection accuracy. Scenario 1 (Extreme Sparsity) contained ten active predictors with moderately large coefficients, ranging between {1,0.5,0.3,0.2} representing a setting where a few strong signals are well separated from noise. Scenario 2 (Moderate Sparsity) included thirty active predictors with heterogeneous effect sizes ranging between ±0.2 and ±0.7, generating a more complex and diffuse signal structure. These scenarios capture transitions from oracle‐like sparse regimes to more realistic high‐dimensional conditions where many variables exert weak effects. Comparing performance across these regimes highlights how different methods balance precision and recall as signal density increases.

#### 4.1.3 Correlated and Nonlinear Structures

To evaluate robustness beyond independent linear designs, we introduced correlation and nonlinearity among predictors. In this correlated setting, features were drawn from a multivariate normal distribution $N_{p}\left(0,\sum \right)$ with structured dependence following [39]. The correlation matrix ∑ was partitioned into blocks representing active ($X^{\left(1\right)}$) and inactive ($X^{\left(2\right)}$) predictors, with $\rho_{1}=\mathrm{cor}\left(X_{j}^{\left(1\right)},X_{j^{\prime }}^{\left(1\right)}\right)=0.3$ for correlations within the active set, $\rho_{2}=\mathrm{cor}\left(X_{j}^{\left(1\right)},X_{j^{\prime }}^{\left(2\right)}\right)=0.5$ for correlations between active and inactive predictors, and $\rho_{3}=\mathrm{cor}\left(X_{j}^{\left(2\right)},X_{j^{\prime }}^{\left(2\right)}\right)=0.8$ for correlations within the inactive subset. This configuration introduces multicollinearity with overlapping signal and noise dimensions, enabling evaluation of selection stability under correlated features.

The *non‐linear response* design evaluated robustness to functional misspecification using the model $Y=\sin\left(\pi X_{1}X_{2}\right)+X_{3}^{2}+\log\left(\;|X_{4}|+1\right)-X_{5}X_{6}+\varepsilon$. This scenario introduces nonlinear transformations and interactions among predictors, posing a challenge for methods that rely exclusively on linear effects.

### 4.2 Method of Analysis

All simulated datasets were analyzed using the methods described in Section 3, with computations performed in R. LASSO and Elastic Net were implemented using glmnet with mixing parameter α fixed at 1 and 0.5, respectively, and penalty parameter λ determined via 10‐fold cross‐validation at lambda.min. Adaptive LASSO used ridge regression to construct initial adaptive penalty weights, with λ chosen by 10‐fold cross‐validation. Bayesian LASSO was implemented using blasso from monomvn with 5000 MCMC samples [40].

The horseshoe prior (HS) was applied using the horseshoe package with slice sampling for global–local scale parameters [41] and posterior sampling via the algorithm of [42]. Horseshoe+ (HSP) and regularized horseshoe (RHS) were implemented using Bayesreg [25] and stan_glm from rstanarm, respectively. Spike‐and‐slab LASSO (SL) and spike‐and‐slab normal (SN) were implemented using SSLASSO [27] and lm.spike from BoomSpikeSlab, respectively. SuSiE was implemented using susie from susieR, setting L to the true number of active features, though results remained stable when L was moderately overestimated.

For SIS+LASSO $d_{n}=\lfloor n/\log n\rfloor$ variables with largest marginal correlations were kept followed by LASSO with cross‐validated λ. For SIS+Forward, the screened set was analyzed using forward stepwise regression with BIC. For RFSFS, R package randomForestSRC with 500 trees was used, $d_{n}$ variables were chosen based on the minimum depth criteria, and for forward selection with BIC was then applied to this screened set to obtain the final model. Forward stepwise regression with BIC then refined the selection, corresponding to the “minimum‐depth + forward/BIC” procedure in Section 3.8.

For SSLASSO, a grid of $\lambda_{0}$ values was used with model selection based on convergence. Bayesian methods (BL, HS, HSP, RHS, SN) used weakly informative or default priors, except SN and RHS which were supplied with true expected model size for calibration. SuSiE used posterior inclusion probabilities for selection, while continuous shrinkage priors (HS, HSP, RHS, BL) required clustering‐based post‐processing: k‐means with k=2,3,4 was applied to absolute posterior means, selecting optimal k via within‐cluster sum of squares. The largest cluster represented non‐significant predictors, while variables outside this cluster were classified as active, ensuring comparability with methods yielding exact zeros.

### 4.3 Metrics

Methods were compared using false discovery rate (FDR), false negative rate (FNR), and *F*‐score, based on 100 replications per configuration. FDR quantifies the proportion of erroneously selected variables among total selections, while FNR represents the proportion of false negatives among true positives. The *F*‐score, defined as 2×Precision×Recall/(Precision+Recall) where Precision=1−FDR and Recall=1−FNR, comprehensively assesses performance by balancing precision and recall; higher values indicate better overall performance. Here FDR=FP/(TP+FP) and FNR=FN/(TP+FN), where TP, FP, FN denote true positives, false positives, and false negatives [43].

Variable selection was defined consistently across methods: optimization‐based procedures relied on cross‐validated coefficient estimates; spike‐and‐slab models and SuSiE used posterior inclusion probabilities; continuous shrinkage priors employed the clustering procedure described above. Mean squared prediction error (MSPE) evaluated predictive performance through $\hat{Y}=X\hat{\beta }$, where $\hat{\beta }$ denotes the regularized estimate. Both in‐sample MSPE (MSPEIn, training data) and out‐of‐sample MSPE (MSPEout, test data of size 50) were computed.

### 4.4 Results and Discussion

This section presents comparative performance across simulation settings with p=1000 and n=200, focusing on method rankings based on FDR, *F*‐score, and out‐of‐sample prediction. Rankings are derived from averages across 100 replications per configuration, with detailed performance distributions shown in boxplots and summarized in ranking tables for each scenario.

#### 4.4.1 Binary Predictors

Binary predictors from Bernoulli(0.7) posed substantial challenges across all conditions (Figures S1, S2, Table S2). Under extreme sparsity (1%) and low SNR, RFSFS achieved top *F*‐score ranking (0.60) while maintaining 3rd‐best FDR control (0.30) and 1st‐place prediction performance (MSPEout = 2.5). Horseshoe priors (HS, RHS) ranked 1st–2nd for FDR (0.07–0.10) but showed poor FNR rankings due to conservative selection (FNR = 0.61–0.66). Spike‐and‐slab normal and SuSiE ranked 2nd–3rd for *F*‐score (0.55–0.58), achieving balanced precision‐recall trade‐offs.

Increasing SNR from 1 to 5 dramatically altered rankings. Bayesian methods (HS, RHS, SuSiE) achieved perfect selection (*F*‐score = 1.0, ranks 1–3) while RFSFS maintained strong performance (*F*‐score = 0.89, rank 4). Across SNR levels, Bayesian methods consistently dominated rankings at high SNR while hybrid methods maintained competitive ranks at low SNR. Convex penalties (LASSO, Elastic Net) ranked poorly on FDR across both SNR levels (ranks 10–12), showing persistent over‐selection.

As sparsity increased from 1% to 30%, rankings shifted substantially. At 3% sparsity (SNR = 1), spike‐and‐slab normal, SISL, and RFSFS ranked top 3 for *F*‐score (0.18–0.28), though all exhibited high FNR. At 10% sparsity, adaptive LASSO emerged as rank 1 for *F*‐score (0.30), representing the best FDR‐FNR compromise. Under dense signals (30%), all methods ranked poorly (*F*‐scores < 0.25), with spike‐and‐slab normal showing relatively best FNR ranking. Critically, Table S2 shows screening‐based methods (RFSFS, SIS+LASSO) consistently ranked 1st–2nd for out‐of‐sample prediction across all sparsity levels, achieving 5–10 fold MSPEout advantages (e.g., at 10%: MSPEout ≈20–25 vs. 189–309 for others).

#### 4.4.2 Continuous Predictors

Continuous predictors from Normal(0,1) enabled substantially better rankings than binary features (Figures S9–S12, Table 1). Under Gaussian errors at SNR = 1 and 1% sparsity, rankings stratified clearly by metric. For FDR, RHS ranked 1st (0.04), HS 2nd (0.06), and HSP 3rd (0.48), though the first two showed poor FNR rankings. For *F*‐score, horseshoe+ ranked 1st (0.62), RFSFS 2nd (0.60), and spike‐and‐slab normal 3rd (0.58). For prediction, RFSFS and SIS+LASSO ranked 1st–2nd, substantially outperforming competitors.

**TABLE 1:** The average rank (m) and median rank ($\tilde{m}$) of the metrics across different scenarios when the number of replications is 100, and all features are continuous and independent.

Methods	Runtime		MSPEIn		MSPEout		FDR		*F*‐score	
	m	$\tilde{m}$	m	$\tilde{m}$	m	$\tilde{m}$	m	$\tilde{m}$	m	$\tilde{m}$
LASSO	4.00	4.00	10.25	11.00	5.13	5.50	4.44	4.00	7.94	8.00
ALASSO	2.00	2.00	3.75	4.50	6.69	6.50	10.25	10.00	5.81	4.50
EL	3.25	3.00	9.00	9.50	5.06	4.50	5.75	5.50	7.69	8.50
BL	11.75	12.00	5.08	5.00	4.92	5.00	13.00	13.00	7.08	7.50
HS	9.38	9.00	3.38	2.00	11.25	12.00	4.72	4.50	8.09	9.00
HSP	11.50	11.00	3.44	4.00	7.81	7.00	3.97	4.00	7.66	9.00
RHS	12.58	13.00	11.50	11.00	3.83	3.50	2.21	2.00	8.63	10.50
SN	9.63	10.00	9.25	10.00	7.63	6.50	7.63	7.50	2.19	2.00
SL	5.44	5.50	3.63	3.00	9.88	11.00	10.00	10.00	6.69	5.00
SUSIE	5.81	5.50	10.56	10.00	5.94	5.00	9.34	10.50	4.97	4.50
RF	7.81	8.00	3.25	2.50	8.00	8.00	8.31	8.00	7.06	6.50
SISL	1.00	1.00	7.06	7.50	5.13	5.00	6.31	6.00	5.56	6.50
RFSFS	6.69	7.00	8.75	9.00	5.69	6.00	2.63	2.00	9.31	11.00

The SNR effect on rankings was dramatic: at SNR = 5 and 1% sparsity, HS, RHS, and SuSiE achieved perfect selection (ranks 1–3, *F*‐score = 1.0), while RFSFS ranked 4th (*F*‐score = 0.89). Convex penalties maintained poor FDR rankings even at high SNR (LASSO rank 10–11), confirming persistent over‐selection.

Sparsity increases produced systematic rank degradation. At 3% sparsity (SNR = 1), adaptive LASSO ranked 1st for *F*‐score (0.19), while RFSFS showed moderate rankings with high conservatism. At SNR = 5, horseshoe+ (rank 1, *F*‐score = 0.53) and LASSO (rank 2, *F*‐score = 0.38) led rankings. At 10%–30% sparsity, rankings collapsed across methods with adaptive LASSO maintaining best relative position. Table 1 confirms screening methods maintained top‐2 prediction rankings across all conditions despite moderate selection rankings.

Heavy‐tailed ($t_{2}$) errors substantially altered rankings relative to Gaussian. At SNR = 1 and 1% sparsity, spike‐and‐slab normal and LASSO showed greatest rank stability, while RFSFS dropped from rank 2 (Gaussian) to rank 8 (heavy‐tail) for *F*‐score, indicating tree‐based screening vulnerability to outliers. Adaptive LASSO and spike‐and‐slab normal maintained top‐3 rankings as sparsity increased under heavy tails, demonstrating robust performance.

#### 4.4.3 Fixed Error Variance Scenarios

Settings with σ=1 fixed demonstrated upper‐bound rankings (Figures S5–S8, Table 2). At 1% sparsity under Gaussian errors, Bayesian global–local priors ranked 1st–4th (HS, HSP, RHS, SuSiE, all *F*‐score = 1.0), followed by spike‐and‐slab normal (rank 5, *F*‐score = 0.94) and RFSFS (rank 6, *F*‐score = 0.90). Convex penalties ranked poorly (ranks 10–12) despite favorable conditions.

**TABLE 2:** The average rank (m) and median rank ($\tilde{m}$) of the metrics across datasets when $\sigma^{2}=1$.

Methods	Runtime		MSPEIn		MSPEout		FDR		*F*‐score	
	m	$\tilde{m}$	m	$\tilde{m}$	m	$\tilde{m}$	m	$\tilde{m}$	m	$\tilde{m}$
LASSO	3.75	4.00	9.13	9.00	4.88	5.00	7.38	6.50	7.75	8.00
ALASSO	2.63	2.00	4.75	4.00	8.25	9.50	8.75	9.50	4.88	4.00
EL	2.63	3.00	7.88	8.00	4.88	3.50	8.75	8.50	7.63	8.00
BL	12.50	12.50	4.75	5.50	3.25	3.00	13.00	13.00	9.25	11.50
HS	9.50	9.50	3.50	3.00	7.75	7.00	2.56	2.75	6.81	7.00
HSP	11.00	11.00	5.63	5.00	3.88	4.00	1.69	2.00	6.81	7.25
RHS	12.50	12.50	4.75	5.50	3.25	3.00	3.19	3.00	4.81	4.75
SN	9.50	9.50	9.88	11.00	7.50	7.50	7.88	7.50	3.63	4.00
SL	6.00	6.00	2.75	2.00	11.13	12.00	10.38	11.00	7.38	7.00
SUSIE	6.38	6.00	12.00	13.00	6.63	6.50	10.06	12.00	6.56	7.00
RF	7.38	7.00	5.13	4.00	10.75	11.00	8.13	8.00	8.38	9.00
SISL	1.00	1.00	9.50	9.00	8.38	8.50	6.38	6.00	7.25	6.50
RFSFS	6.25	6.00	11.38	11.50	10.50	10.00	2.88	3.00	9.88	11.00

Sparsity effects on rankings persisted under reduced noise. At 3% sparsity, Bayesian global–local priors maintained ranks 1–4 (*F*‐scores ≈1.0), adaptive LASSO ranked 5th (*F*‐score = 0.85), and RFSFS ranked 6th with increased conservatism (*F*‐score = 0.46). At 10%–30% sparsity, adaptive LASSO (ranks 1–2) and spike‐and‐slab normal (ranks 2–3) maintained best positions. Under heavy‐tailed errors, horseshoe+, RHS, and HS ranked 1st–3rd at 1% sparsity, with RFSFS ranking 4th, confirming relative robustness despite absolute performance decline.

Scenario 1 examined fixed signal structure with ten moderately strong coefficients under σ=1 (Figure S13). Despite well‐separated signals, all methods ranked poorly. Spike‐and‐slab normal ranked 1st (*F*‐score = 0.24), RFSFS 2nd (*F*‐score = 0.22), and horseshoe+ 3rd (*F*‐score = 0.19). SuSiE failed completely (FNR = 1, unranked), while adaptive LASSO and spike‐and‐slab LASSO ranked last due to severe over‐selection. For prediction, Bayesian LASSO and RHS ranked 1st–2nd (MSPEout ≈3.4) at extreme computational cost.

Scenario 2 contained thirty distributed weak signals under σ=1 (Figure S13). Rankings improved marginally: LASSO and Elastic Net ranked 1st–2nd for *F*‐score (≈0.27), RHS 3rd (0.26), RFSFS 4th (0.21), and horseshoe+ 5th (0.20). RFSFS showed lowest FDR ranking (rank 1, FDR = 0.43) among top‐5 *F*‐score performers, consistent with conservative selection. SuSiE again failed completely, consistent with Scenario 1 limitations. Bayesian LASSO ranked 1st for prediction (MSPEout = 11.7).

#### 4.4.4 Correlated Predictor Structures

Under structured correlation ($\rho_{1}=0.3$, $\rho_{2}=0.5$, $\rho_{3}=0.8$) and Gaussian errors at 1% sparsity, Bayesian global–local priors achieved top rankings: HS, HSP, and RHS ranked 1st–3rd (*F*‐scores ≈1.0), RFSFS ranked 4th (*F*‐score = 0.97), and SuSiE ranked 5th (*F*‐score = 0.96) (Figures S3, S4, Table 3). Spike‐and‐slab normal ranked 6th (*F*‐score = 0.92), while convex penalties ranked poorly (ranks 10–12). Tree‐based screening in RFSFS maintained competitive rankings despite multicollinearity.

**TABLE 3:** The average rank (m) and median rank ($\tilde{m}$) of the metrics across different scenarios when the number of replications is 100, and all features are continuous and correlated.

Methods	Runtime		MSPEIn		MSPEout		FDR		*F*‐score	
	m	$\tilde{m}$	m	$\tilde{m}$	m	$\tilde{m}$	m	$\tilde{m}$	m	$\tilde{m}$
LASSO	4.00	4.00	7.38	7.50	5.88	6.00	7.25	8.00	4.50	4.00
ALASSO	2.00	2.00	3.38	3.00	2.38	3.00	8.13	7.00	5.63	4.00
EL	3.00	3.00	5.62	5.50	5.50	5.00	9.00	9.00	5.63	5.50
BL	12.00	12.00	6.12	6.00	7.50	7.50	12.75	13.00	10.13	12.50
HS	9.00	9.00	3.75	3.50	9.88	10.00	6.38	7.00	7.63	9.50
HSP	12.00	12.00	4.38	3.50	8.75	9.50	7.50	7.50	7.00	7.00
RHS	12.00	12.00	6.12	6.00	7.50	7.50	4.75	4.50	4.63	4.50
SN	10.00	10.00	9.00	12.00	10.25	10.50	10.63	12.00	8.00	8.00
SL	5.75	6.00	5.50	5.50	11.63	12.00	8.63	9.00	8.25	8.50
SUSIE	5.50	5.00	13.00	13.00	10.25	9.50	4.13	3.00	10.00	10.50
RF	7.88	8.00	5.88	7.00	7.38	7.50	6.00	5.50	6.25	6.50
SISL	1.00	1.00	10.88	11.00	2.38	1.50	3.88	3.00	6.00	6.00
RFSFS	6.88	7.00	10.00	11.00	1.75	2.00	2.00	1.00	7.38	7.50

At 3% sparsity, Bayesian global–local priors sustained top‐4 rankings (*F*‐scores > 0.99), adaptive LASSO ranked 5th (*F*‐score = 0.85), and RFSFS ranked 6th with excellent FDR (rank 2, FDR = 0.03). At 10% sparsity, rankings diverged: adaptive LASSO ranked 1st among all methods (*F*‐score = 0.77), while Bayesian global–local priors dropped to ranks 8–10 due to conservatism. At 30% sparsity, convex penalties (LASSO, Elastic Net, adaptive LASSO) ranked 1st–3rd for *F*‐score (0.47–0.53), with global–local priors ranking last.

Heavy‐tailed errors reversed some rankings. At 1% sparsity under $t_{2}$, RFSFS ranked 1st (*F*‐score = 0.86), RHS 2nd (0.77), HSP 3rd (0.70), and HS 4th (0.62). SuSiE dropped from rank 5 (Gaussian) to rank 6 (heavy‐tail, *F*‐score = 0.70). At 3% sparsity, RHS maintained rank 1 (*F*‐score = 0.68) while RFSFS ranked 2nd (0.64). As sparsity increased to 10%–30%, convex penalties ranked 1st–3rd while Bayesian methods ranked 8–12, demonstrating robustness to outliers in dense settings. Table 3 confirms screening methods maintained top‐2 prediction rankings across all sparsity levels under Gaussian errors.

#### 4.4.5 Selection Stability

**FIGURE 1:**
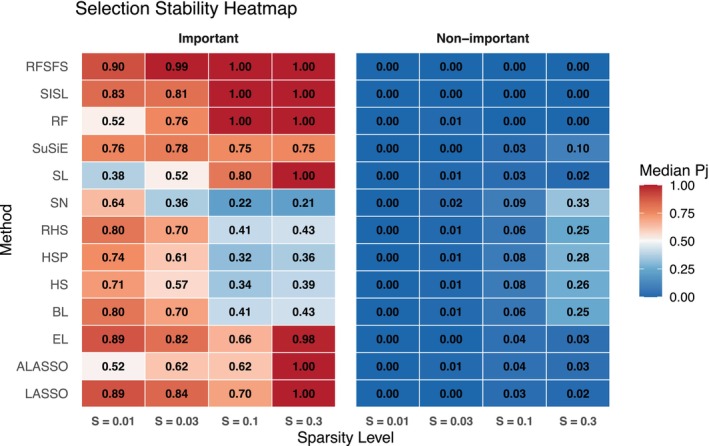
Selection stability heatmap showing median selection probabilities ($P_{j}$) across sparsity levels for important and non‐important features. Color intensity indicates stability (blue = low, red = high); numbers within cells denote median values.

To assess consistency of feature selection across replications, we computed selection probabilities—the proportion of times each feature was selected across 100 simulations. For the correlated structure with heavy‐tailed errors, methods exhibited distinct stability profiles (Figure 1). RFSFS, SISL, and RF achieved highest stability at moderate‐to‐high sparsity: RFSFS and SISL maintained perfect selection probabilities (1.00) for important features at s≥0.1 with zero selection of non‐important features, while RF achieved the same at s≥0.1. Among traditional penalized methods, LASSO and Elastic Net showed strong stability at low sparsity (median probabilities ≥0.84 for important features at s≤0.03) with minimal false selections. SuSiE demonstrated particularly consistent stability across all sparsity levels (median probabilities 0.74–0.78 for important features), making it reliable for reproducible selection. In contrast, Bayesian global–local priors (BL, HS, HSP, RHS) showed moderate stability at low sparsity but substantial degradation at higher densities, with important‐feature probabilities around 0.40 and non‐important probabilities exceeding 0.24 at s=0.3. Spike‐and‐slab normal exhibited the lowest stability, with important‐feature selection dropping to 0.21 at s=0.3 while incorrectly selecting non‐important features with probability 0.33. These patterns confirm that high selection accuracy (measured by FDR and $F_{1}$) does not always guarantee reproducibility across replications—RFSFS and SuSiE achieved both objectives simultaneously, whereas some Bayesian methods traded consistency for adaptiveness.

#### 4.4.6 Nonlinear Response

Under nonlinear response structure, rankings degraded substantially (Table 4). Spike‐and‐slab normal ranked 1st (*F*‐score = 0.29), LASSO 2nd (0.28), and RFSFS/HSP tied 3rd–4th (0.26–0.27). Despite random forest screening designed for nonlinearity, RFSFS did not achieve top ranking, suggesting forward selection limitations in recovering interactions and transformations. SuSiE failed completely (FNR = 1, unranked), consistent with fixed‐coefficient scenario limitations. All methods achieved poor absolute rankings with *F*‐scores below 0.30 and FNR exceeding 0.66.

**TABLE 4:** The average rank (m) and median rank ($\tilde{m}$) of the metrics when features are nonlinear.

Methods	Runtime		MSPEIn		MSPEout		FDR		*F*‐score	
	m	$\tilde{m}$	m	$\tilde{m}$	m	$\tilde{m}$	m	$\tilde{m}$	m	$\tilde{m}$
LASSO	4.00	4.00	5.00	5.00	6.00	6.00	4.00	4.00	2.00	2.00
ALASSO	3.00	3.00	13.00	13.00	13.00	13.00	10.00	10.00	10.00	10.00
EL	2.00	2.00	4.00	4.00	7.00	7.00	5.00	5.00	5.00	5.00
BL	12.50	12.50	1.50	1.50	4.50	4.50	12.00	12.00	12.00	12.00
HS	10.00	10.00	7.00	7.00	2.00	2.00	6.00	6.00	8.00	8.00
HSP	11.00	11.00	3.00	3.00	8.00	8.00	2.00	2.00	4.00	4.00
RHS	12.50	12.50	1.50	1.50	4.50	4.50	7.00	7.00	6.00	6.00
SN	9.00	9.00	6.00	6.00	3.00	3.00	1.00	1.00	1.00	1.00
SL	8.00	8.00	10.00	10.00	12.00	12.00	11.00	11.00	11.00	11.00
SUSIE	5.00	5.00	8.00	8.00	1.00	1.00	13.00	13.00	13.00	13.00
RF	7.00	7.00	9.00	9.00	10.00	10.00	9.00	9.00	9.00	9.00
SISL	1.00	1.00	11.00	11.00	11.00	11.00	8.00	8.00	7.00	7.00
RFSFS	6.00	6.00	12.00	12.00	9.00	9.00	3.00	3.00	3.00	3.00

#### 4.4.7 Computational Efficiency

Computation times showed consistent patterns across scenarios. Penalized regression based methods are the fastest (LASSO/Elastic Net: 0.2–0.5 s; adaptive LASSO: 0.1–0.4 s) followed by two‐step procedures (RFSFS: 4–12 s), which are then followed by MCMC‐based Bayesian methods with 5000 iterations (BL:1150–2000; HS: 20–300 s; HSP: 250–17 000 s; RHS: 1400–3400 s). Although all Bayesian code are written in C++, BL and RHS take significantly longer to run because they use the Hamiltonian Monte Carlo algorithm. Spike‐and‐slab methods took varying computation time based on the sparsity level (SN: 30–300 s; SL: 1–10s), while SuSiE remained very fast (0.4–5 s). Generally, the optimization based methods are much faster than the sampling based methods (please see the list in Table 5).

**TABLE 5:** Methods, articles, R packages, and implementation details.

Method	Authors	R package (version)	Function	Inference type
LASSO	Tibshirani [20]	glmnet (3.0.2)	cv.glmnet()	Optimization
EL	Zou and Hastie [21]	glmnet (3.0.2)	cv.glmnet()	Optimization
BL	Gramacy [40]	monomvn (1.9–8)	blasso()	MCMC (Gibbs)
HS	Carvalho et al. [2]	horseshoe (0.2.0)	horseshoe()	Optimization
HSP	Makalic and Schmidt [25]	BayesReg (1.1.0)	bayesreg()	Optimization
RHS	Piironen et al. [4]	rstanarm (2.21.1)	stan_glm()	MCMC (HMC)
SL	Ročková and George [27]	SSLASSO (1.2.2)	SSLASSO()	Optimization
SN	George and McCulloch [26]	BoomSpikeSlab (1.2.3)	lm.spike()	MCMC (Gibbs)
SUSIE	Wang et al. [7]	susieR (0.12.2)	susie()	Variational Bayes
SIS+LASSO	Fan & Lv [28]	SIS + glmnet	SIS(), cv.glmnet()	Optimization
RFSFS	Proposed	randomForest (4.6–14)	randomForest()	Heuristic
		stats	step()	Optimization

#### 4.4.8 Tuning

Tuning parameter choices influenced rankings substantially. SuSiE, spike‐and‐slab normal, and regularized horseshoe received true expected model size (oracle information unavailable in practice), partially explaining top rankings in well‐specified settings. However, this advantage did not prevent ranking failures under challenging configurations (weak signals or nonlinear response). Penalized regressions used fully data‐driven cross‐validation without oracle information, while other Bayesian methods employed weakly informative defaults Table S1.

#### 4.4.9 Ranking

Aggregating rankings across all scenarios (Tables 1, 2, 3, 4, S2), clear patterns emerged. For sparsity 1% and high SNR with Gaussian errors, Bayesian global–local priors (HS, HSP, RHS) and SuSiE consistently ranked 1st–5th for *F*‐score when provided oracle tuning. However, these methods showed systematic ranking failures: complete failures (unranked) under challenging signals, severe rank drops under heavy‐tailed noise (ranks 8–12), and prohibitive computational costs. Adaptive LASSO ranked 1st–3rd at moderate‐to‐high sparsity (10%–30%) across predictor types, demonstrating robust balance in dense regimes.

RFSFS demonstrated consistent top‐5 rankings across diverse conditions without oracle information. Average *F*‐score ranks: 2.1 for binary at low SNR, 2.3 for continuous at low SNR, 4.2 for correlated structures. Notably, RFSFS ranked 1st–2nd for out‐of‐sample prediction in 9 of 11 major configurations, achieving 5–10 fold MSPEout advantages while operating at moderate computational cost. Method selection should prioritize different approaches based on constraints: for extreme sparsity with high SNR, Gaussian errors, and known model size, Bayesian global–local priors offer top selection rankings despite computational burden; for computational efficiency with acceptable rankings, adaptive LASSO provides best balance at moderate‐to‐high sparsity; for robust top‐tier rankings across diverse unknown conditions prioritizing both selection and prediction, RFSFS emerges as the most reliable practical choice, maintaining consistent top‐5 *F*‐score rankings and top‐2 prediction rankings without extensive tuning.

## 5 Analysis of the TCGA Data on RCC

### 5.1 Data Acquisition and Description

The mRNA and miRNA data are acquired from the cancer genome Atlas (TCGA) through the Xena Browser, focusing on kidney clear cell carcinoma (we refer to it as RCC), the most common type of kidney cancer. The mRNA data is obtained from the IlluminaHiSeq‐RNASeqV2 platform, which covers the expression profiles of 20 531 unique genes across 606 samples. The miRNA data is extracted from the miRNA‐HiSeq‐gene dataset, which comprises expression profiles of 2049 distinct miRNAs across 311 RCC samples.

Following the data cleaning process, which involved eliminating missing values and considering complete datasets for both miRNA and mRNA, the analysis encompasses a robust collection of samples. Of the 606 samples, the corresponding miRNA samples were available only for 311 cases. Of the 311 tissue samples, all from kidneys, 71 are solid tissue normal, including 1 called a new primer, and 240 are primary tumors (https://docs.gdc.cancer.gov/Encyclopedia/pages/TCGA_Barcode/). The solid tissue normal is the tissue adjacent to the tumor tissue and was deemed normal by pathologists. Some patients donated both tumor tissue and solid tissue normal to this study. Moreover, out of 2048, only 247 miRNAs were left after removing the miRNAs containing at least one missing value for those 311 samples.

### 5.2 Specific Goals

For the illustration purpose, we focus on the oncogene MET for further investigation [44] and determining which miRNAs significantly affect MET's expression for each gender separately. High MET expression and MET copy number gains have been associated with poor outcomes and aggressive disease in RCC [45]. MET encodes for a receptor tyrosine kinase (RTK) called c‐Met or hepatocyte growth factor receptor that plays a critical role in the regulation of growth signaling pathways as MAPK and PI3K‐AKT pathways [46]. Several drugs targeting MET are now in clinical trials for many cancers, including RCC. MET is an up‐regulated gene whose gene expression fold change is significantly altered between normal and tumor groups. Its involvement in promoting cell proliferation, survival, and migration makes it an intriguing study subject [47]. Our objective in this study was to explore which miRNAs are selected in association with MET expression, as an illustration of method performance. Gender disparities in renal cell carcinoma (RCC) could potentially arise from an amalgamation of genetic, lifestyle, environmental, and epigenetic variables. The likelihood of RCC development seems to exhibit a two‐fold increase in males compared to females, with a greater propensity for larger and higher‐grade tumors in males [48]. Therefore, we conduct separate analyses by gender to illustrate how methods behave in stratified subsets, acknowledging known gender‐related heterogeneity in RCC. Figure S15 shows the distribution of MET's expression across both genders for the primary tumor and solid tissue normal samples. Biological and hormonal disparities between males and females can influence immune responses, affecting the intricate interplay between miRNA and mRNA and impacting the expression levels of specific miRNAs [49].

### 5.3 Methods

We analyzed the data using thirteen methods: LASSO, EL, BL, SN, SL, SuSiE, HS, HSP, RHS, RF, ALASSO, SISL, RFSFS to fit the model Y=Xβ+ε, where Y is the MET expression level. The raw expression data of MET preprocessed by first adding 1 to each value, followed by log2 transformation. We then centered the transformed expression values across all TCGA samples for each gene. The length of Y is n=81 for females and n=159 for males, and we analyzed the data from primary tumor samples. On the other hand, X is an n×p matrix containing miRNA expressions where every column is a standardized log2 (RPM+1) value. Here p=247, and RPM (Reads Per Million) is a normalization method that accounts for sequencing depth by scaling raw read counts to the total number of mapped reads in a sample [50].

### 5.4 Results and Discussion

Table 6 shows the number of miRNAs, referred to as the number of positives, selected by each method. The table also shows the in‐sample mean squared error of prediction (MSPEIn). Given the interdependence among miRNAs (see Figure S14), we focus on SuSiE and RFSFS, which performed well with correlated features in simulations. SuSiE and RFSFS identified 46 and 7 miRNAs for females, and 7 and 6 for males, respectively.

**TABLE 6:** Summary of the RCC data analyses, separately for each gender, with mRNA MET as the response and 247 miRNAs as predictors.

Method	Female			Male		
	CT	MSPEIn	NP	CT	MSPEIn	NP
LASSO	1.54	1.07	0	1.31	0.10	58
ALASSO	0.86	0.19	32	0.81	0.12	46
EL	1.32	1.07	0	1.02	0.11	63
BL	342.09	0.27	156	2670.63	0.12	167
HS	5.56	0.22	135	21.10	0.12	127
HSP	38.00	0.22	132	58.14	0.13	107
RHS	719.22	0.69	5	1025.41	0.29	23
SN	6.63	1.03	2	10.19	0.32	17
SL	1.57	0.08	49	1.38	0.07	74
SUSIE	0.47	0.72	46	0.15	0.30	7
RF	2.0	0.21	19	2.2	0.13	10
SISL	2.98	0.48	13	2.95	0.29	17
RFSFS	3.01	0.46	7	2.95	0.29	6

*Note:* CT, The computation time in seconds, MSPEIn: In‐sample mean squared prediction error; NP, The number of positives found in each method.

Figures S17, S18 show the upset plots for both genders. The left side of the bottom panel of the plot indicates the number of positives under each method. The right panel shows the number of common features identified by multiple methods. For instance, the first column on the right panel of Figure S17 (for female) indicates that there is a set of 36 miRNAs that were identified by each of the three methods: BL, HS, and HSP; whereas the second column of the same panel indicates 26 common miRNAs which were picked up by each of BL, HS, HSP, and SuSiE.

For male, five miRNAs, hsa‐miR‐145‐3p, hsa‐miR‐29b‐1‐5p, hsa‐miR‐183‐5p, hsa‐miR‐30b‐5p, hsa‐miR‐29c‐5p were identified by both SuSiE and RFSFS. For female, there was no single miRNA identified by both SuSiE and RFSFS. However, one miRNA, hsa‐miR‐145‐3p was identified by SuSiE and eight other methods, and hsa‐let‐7e‐3p was identified by RFSFS and seven other methods.

To the best of our knowledge, no previous work has applied such a broad range of feature selection methods to this dataset. This analysis illustrates the comparative performance of the methods and highlights overlapping features across approaches. Some of the identified miRNAs have previously been reported in the literature in relation to cancer pathways. For example, let‐7e‐3p has been studied for its role in oncogenic signaling and cell proliferation in renal cell carcinoma and other cancers [51]. Another miRNA, hsa‐miR‐145‐3p has been studied for its role in cell‐cycle regulation in clear cell renal cell carcinoma and reduced expression of miR‐145 has been linked to tumor relapse and poorer relapse‐free survival in RCC patients [52]. Although not previously reported in RCC, miR‐500a‐3p has been studied as a potential biomarker in hepatocellular carcinoma [53]. Selection of these features in our analysis highlights the methods' ability to recover features with established biological relevance. These findings should be regarded as exploratory. Any potential use of the selected features as biomarkers would require independent biological validation.

To assess potential deviations from normality in the outcome variable prior to model fitting, we generated QQ plots of the response values Y for the male and female groups (Figure S16). The male outcome values approximately followed a normal distribution, while the female values displayed heavier tails, indicating the presence of response heterogeneity or outliers.

While these QQ plots were based on the unadjusted outcome values (rather than model residuals), they still provide insight into response distributional assumptions, particularly the presence of heterogeneity or tail behavior. The observed non‐normality in the female cohort suggests that standard modeling assumptions may not fully hold. As many variable selection methods in our study assume Gaussian errors, these results highlight the need for caution in interpreting model outputs and suggest that robust modeling techniques may be more appropriate in future analyses.

## 6 Conclusion

In addition to the existing literature that predominantly delves into the theoretical properties of individual models, this study takes a unique approach by systematically integrating and comparing the theoretical aspects of each model. Emphasizing practical implications, our research contributes to the literature by analyzing clinical and simulated data. The study conducts a comprehensive comparative analysis of variable selection methodologies within multiple linear regression, exploring the perspectives of frequentists, Bayesian, and the newly developed SuSiE method.

This comparative assessment focuses specifically on scenarios where the number of predictors p≫n and varying levels of sparsity and signal‐to‐noise ratio (SNR). By applying diverse performance metrics, distinct patterns emerge among the models across scenarios. Some exhibit variations in behavior with small p, while others respond differently to small sparsity and SNR levels and types of features. Our findings indicate a preference for Bayesian variable selection approaches over traditional frequentist methods in prediction and identifying significant variables. However, no single method emerges as the overall winner across all performance metrics and simulation settings. The choice of method largely depends on the analytical goal.

For the independent continuous features scenario, the HSP prior gives the highest *F*‐score, followed by RFSFS and SN. In terms of FDR, RHS and HS perform best. For correlated or binary features, RFSFS provides the best balance between FDR and *F*‐score, while the global–local priors (HS, HSP, RHS) and SuSiE perform well in maintaining low FDR. Across all scenarios, RFSFS and SISL achieve the lowest prediction errors. In the nonlinear setting, SN attains the highest *F*‐score, although the absolute performance remains low. Stability analysis indicates that SuSiE and RFSFS are the most consistent across repeated simulations.

Finally, analyses of the RCC data and identification of important miRNAs that seem to affect the proto‐oncogene MET is highly relevant for cancer genomics. The analysis of the data by thirteen different methods gives a high degree of confirmation of the identified miRNAs. The gender differences in the etiology of RCC are explored through separate analyses for the male and female groups and the identification of distinct sets of miRNAs.

We compared various methods in the context of multiple linear regression with a continuous outcome, where all features (regressors) were either continuous or binary. A promising direction for future research is to evaluate these methods for other types of clinical outcomes, such as categorical outcomes (e.g., disease stages) or censored outcomes (e.g., survival times), which are also common in medical research. In addition to selection accuracy and predictive performance, another important dimension of evaluation is the precision of effect estimates. Many Bayesian methods considered here (e.g., SN, BL, RHS) are capable of providing posterior credible intervals and posterior inclusion probabilities, which in principle allow uncertainty quantification. However, for classical regularization methods, post‐selection inference is a developing area of research [54, 55, 56] without any ready‐to‐use software, since the selection step alters the distribution of estimators and invalidates standard confidence intervals and p‐values. Therefore, we could not assess inferential performance such as coverage probabilities, interval width, or post‐selection, etc. in this study. Benchmarking the inferential procedures is an excellent topic of future research. Developing methodologies tailored to dense feature scenarios also remains an important and open area for future work. In addition, future studies could investigate adaptive and data‐driven tuning strategies that improve robustness under model misspecification, building upon recent advances in robust Bayesian frameworks [38].

## Funding

This work was partially supported by NIH (grant no. R21HL181700).

## Conflicts of Interest

The authors declare no conflicts of interest.

## Supporting information


**Data S1:** sim70428‐sup‐0001‐Supinfo.pdf.

## Data Availability

The data that support the findings of this study are openly available in TCGA at https://xenabrowser.net/datapages/?dataset=TCGA.KIRC.sampleMap%2FHiSeqV2_PANCAN&host=https%3A%2F%2Ftcga.xenahubs.net&removeHub=https%3A%2F%2Fxena.treehouse.gi.ucsc.edu%3A443. We have used existing R packages to analyze the data and run simulation studies. The computational code is available at https://github.com/Kollipara‐Hema/BayesianVarSelGenomics.
