# Supplementary material for: Benchmarking Sparse Variable Selection Methods for Genomic Data Analyses
Source: Stat Med. 2026 Feb 10;45(3-5):e70428. doi: 10.1002/sim.70428 (PMC12888550; doi:10.1002/sim.70428)
Supplement: Supplementary file 1 — Data S1: sim70428‐sup‐0001‐Supinfo.pdf. [file SIM-45-0-s001.pdf]

Supplementary materials for "Benchmarking Sparse Variable Selection Methods for Genomic Data Analyses"

Hema Kollipara[1,†], Tapabrata Maiti[1], Sanjukta Chakraborty[2] and Samiran Sinha[3]

[1]Department of Statistics & Probability, Michigan State University, East Lansing, MI
[2]Department of Medical Physiology, Texas A&M Health Science Center, College of Medicine, Bryan, TX
[3]Department of Statistics, Texas A&M University, College Station, TX
[†]email: kollipa4@msu.edu

Table S.1: Tuning parameter and prior specifications for all methods.

**Method**	**Tuning/Prior Specification**
LASSO	$\alpha = 1$ (pure ℓ_1 penalty), λ selected via 10-fold cross-validation at lambda.min
ALASSO	Adaptive weights $\omega_j = 1/\lvert\hat{\beta}_j^{\text{ridge}}\rvert$ from initial ridge regression, λ via 10-fold CV
EL	$\alpha = 0.5$ (equal ℓ_1/ℓ_2 mixing), λ selected via 10-fold cross-validation at lambda.min
BL	5000 MCMC iterations (Gibbs sampling), default priors: $\lambda^2 \sim \text{Gamma}(1,1)$, $\sigma^2 \sim \sigma^{-2}$
HS	Global shrinkage $\tau \sim C^+(0,1)$, local shrinkage $\lambda_j \sim C^+(0,1)$, $\sigma^2 \sim \sigma^{-2}$ (weakly informative)
HSP	Package default priors with hierarchical structure: $\lambda_j\lvert\eta_j \sim C^+(0,\eta_j)$, $\eta_j \sim C^+(0,1)$
RHS	**Oracle information**: True expected model size p_0 supplied to set $\tau_0^2 = p_0/(n-p_0)$; global $\tau \sim C^+(0,\tau_0^2)$, regularization $c^2 \sim$ Inverse-Gamma; Hamiltonian Monte Carlo sampling
SL	Grid search across λ_0 values, convergence-based selection along solution path
SN	**Oracle information**: True expected model size supplied; spike variance $\epsilon^2 \approx 0.01$, slab variance $c^2 \approx 100$ (package defaults); selection via posterior inclusion probabilities
SuSiE	**Oracle information**: L set to true number of non-zero signals; effect variances τ_l^2 and probability vectors π_l estimated from data; selection via posterior inclusion probabilities
SIS+LASSO	Sure Independence Screening retains top $\lfloor n/\log n\rfloor$ features by marginal correlation, followed by LASSO with λ selected via 10-fold cross-validation
RFSFS	Random forest with 500 trees for screening via minimum depth criterion, retain top $\lfloor n/\log n\rfloor$ features, then forward stepwise selection with BIC

C^+: Half-Cauchy distribution; CV: Cross-validation.
Oracle information: Methods received true model size (unavailable in practice).

Table S.2: The average rank (m) and median rank ($\widetilde{m}$) of the metrics across different scenarios when the number of replications is 100, and all features are binary and independent.

Methods	Runtime		MSPEIn		MSPEout		FDR		Fscore	
	m	$\widetilde{m}$	m	$\widetilde{m}$	m	$\widetilde{m}$	m	$\widetilde{m}$	m	$\widetilde{m}$
LASSO	3.75	4.00	7.88	8.00	7.75	8.00	6.38	6.00	7.50	8.00
ALASSO	2.00	2.00	2.00	1.50	2.63	3.00	10.00	10.50	5.63	5.00
EL	3.25	3.00	7.25	7.00	7.50	7.00	7.38	7.50	7.38	7.50
BL	11.25	11.00	5.25	6.00	6.50	6.00	13.00	13.00	7.00	7.00
HS	9.50	9.50	5.63	5.00	9.50	9.50	3.25	3.00	7.50	8.50
HSP	12.00	12.00	3.25	3.50	8.63	8.00	3.50	3.00	7.25	9.50
RHS	11.75	12.00	11.00	11.00	6.50	6.00	1.25	1.00	7.75	8.50
SN	9.50	9.50	9.50	10.00	8.50	9.00	7.75	7.50	2.13	2.00
SL	6.38	6.00	2.75	2.00	11.13	11.50	10.75	11.00	7.25	7.50
SUSIE	5.75	5.00	11.88	11.50	8.13	8.00	5.50	5.00	4.50	3.50
RF	7.25	7.50	4.00	3.50	4.88	4.00	8.63	8.50	6.63	6.00
SISL	1.00	1.00	6.38	6.00	1.75	2.00	6.00	6.00	5.75	7.00
RFSFS	6.63	7.00	9.88	9.00	1.63	1.00	2.25	2.00	9.63	11.00

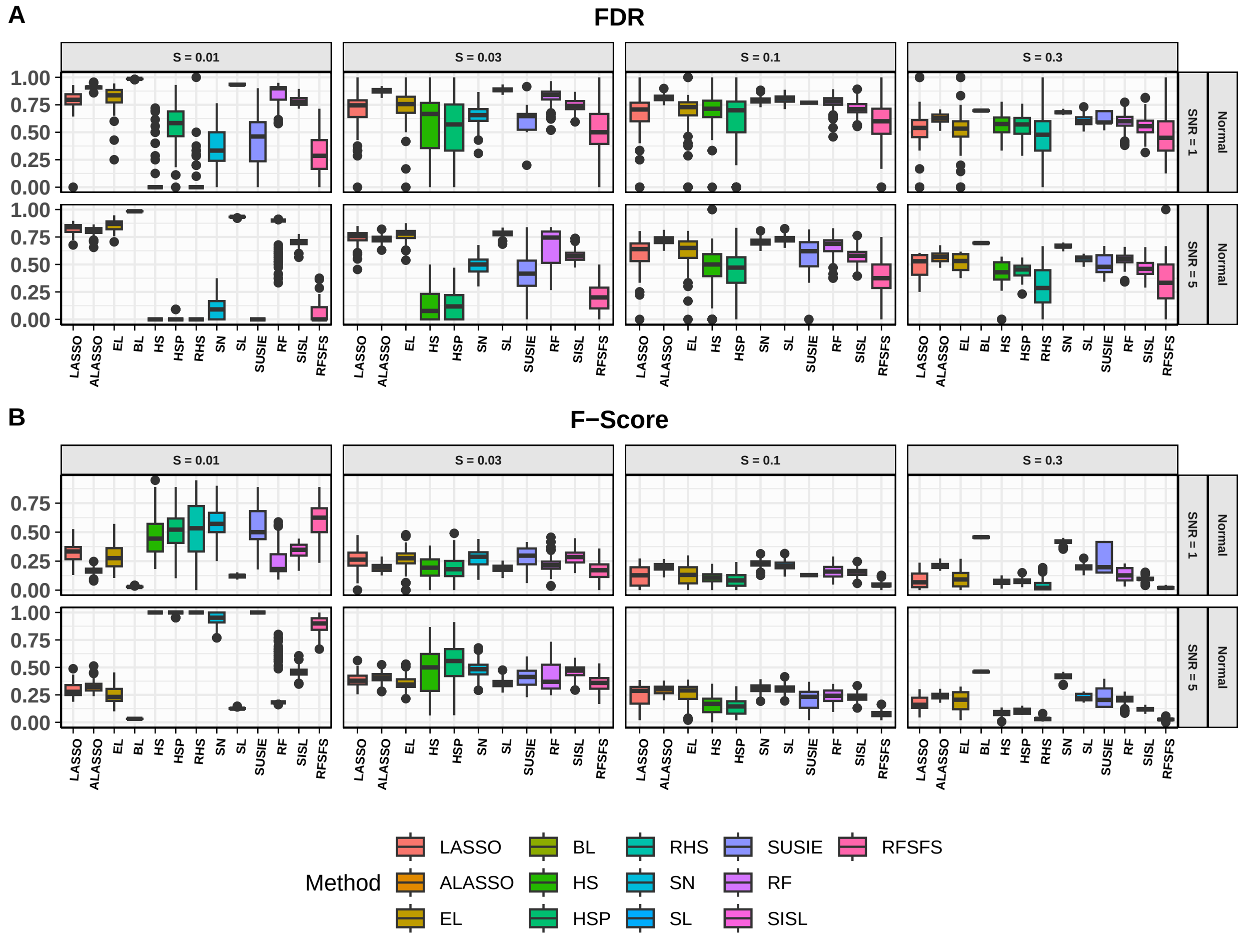

Figure S-1: The plot of FDR, Fscore under different methods and simulation scenarios when the features are binary and independent.

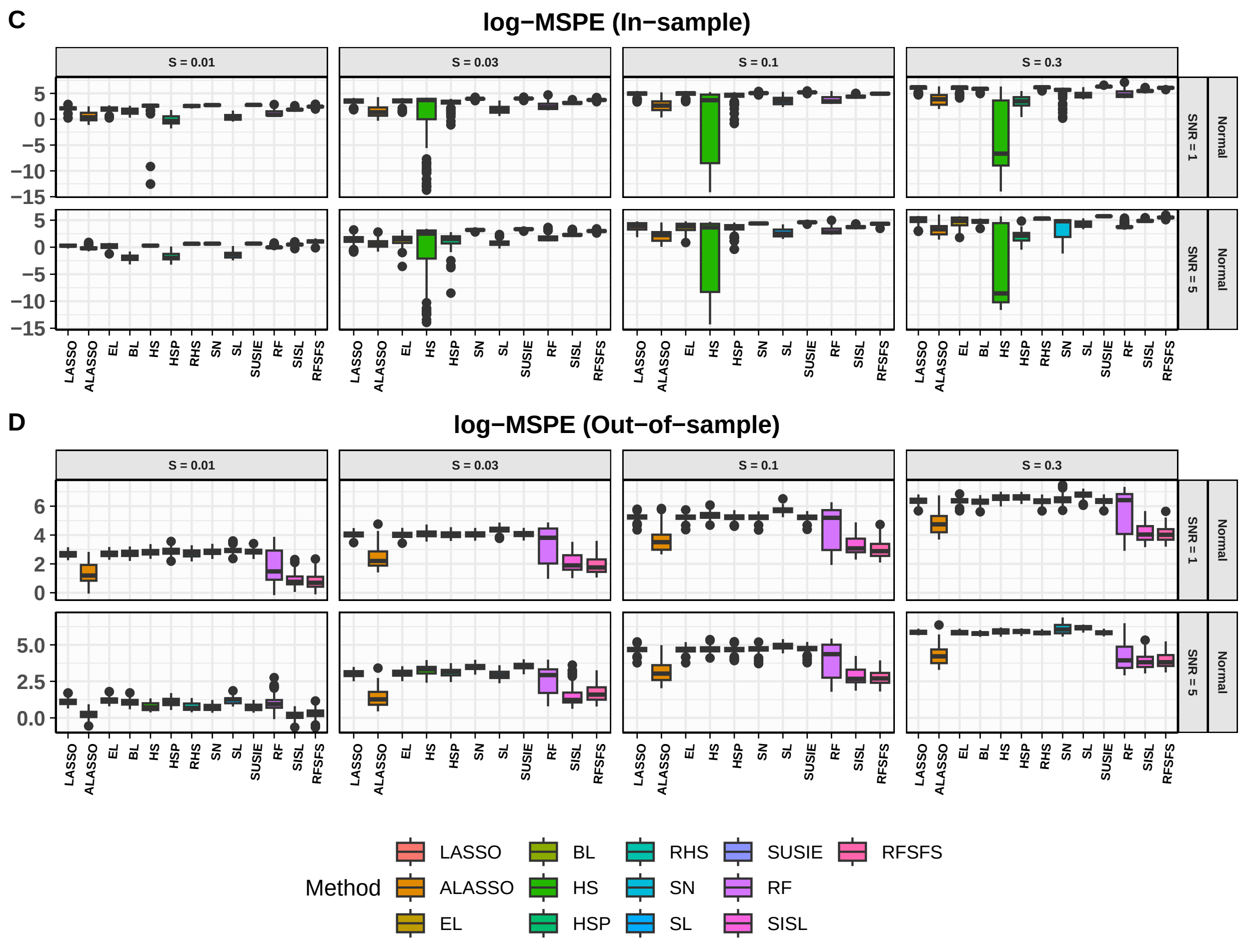

Figure S-2: The plot of MSPEIn, MSPEout under different methods and simulation scenarios when the features are binary and independent.

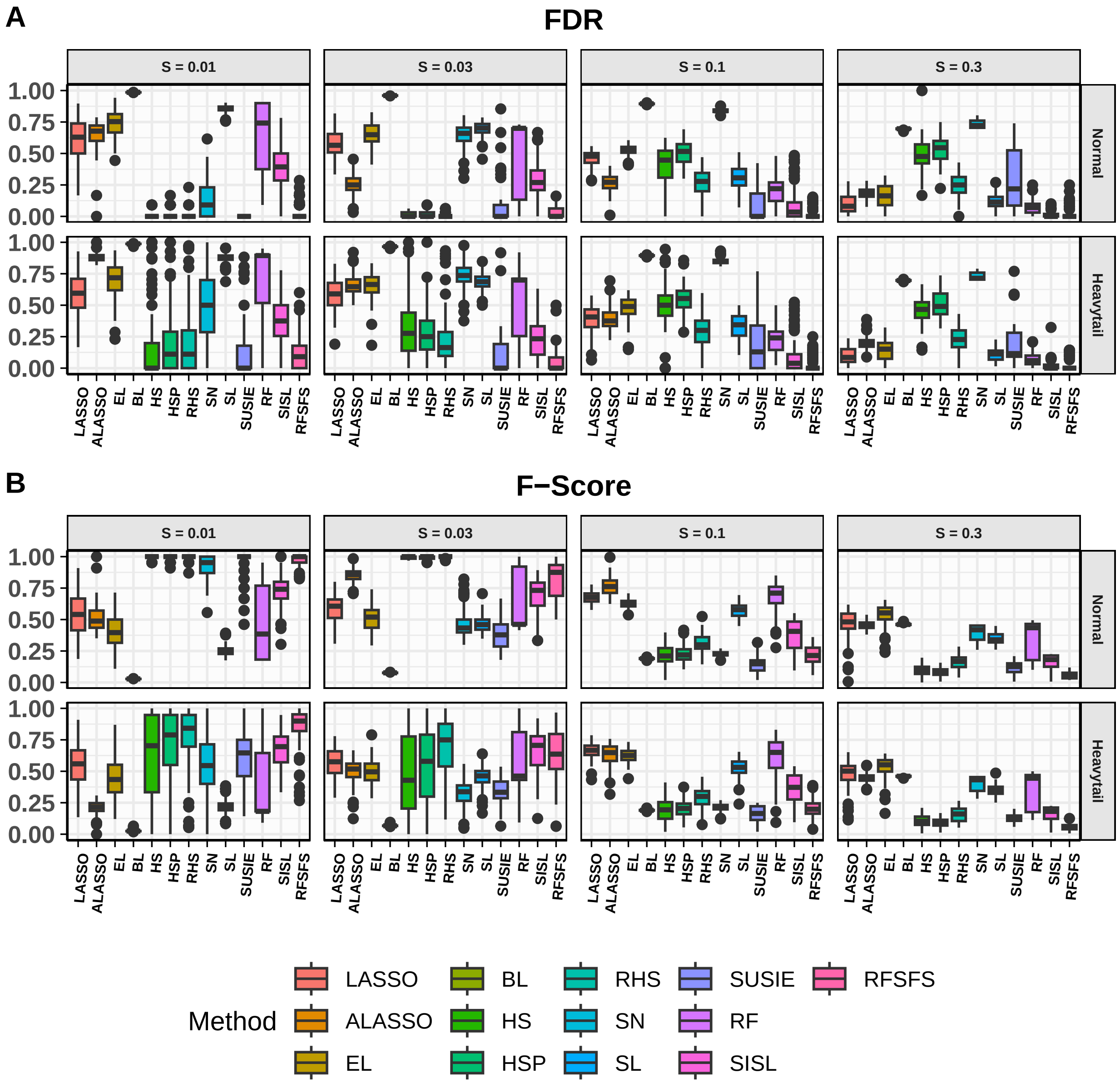

Figure S-3: The plot of FDR, Fscore under different methods and simulation scenarios when the features are continuous and correlated.

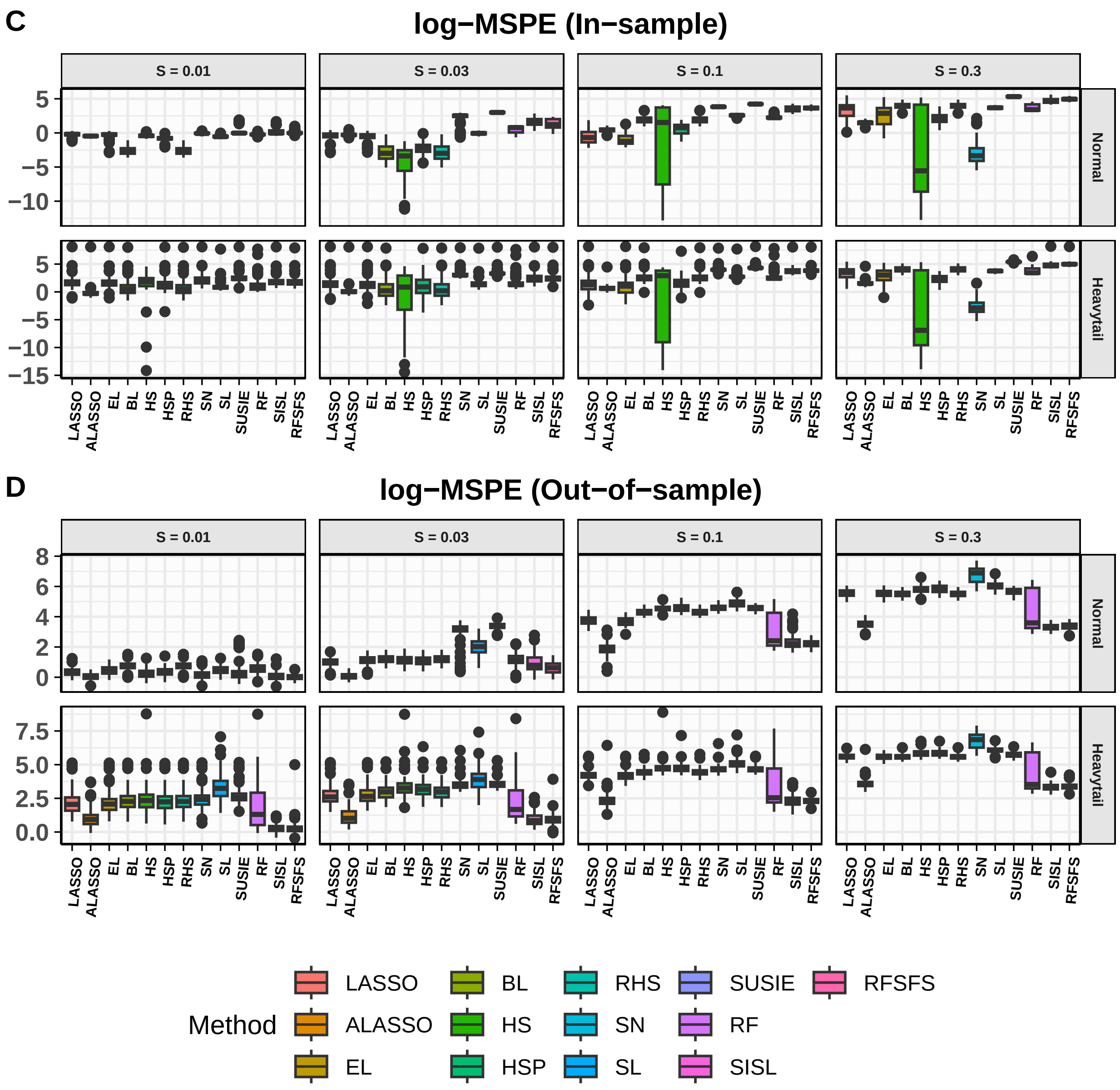

Figure S-4: The plot of MSPEIn, MSPEout under different methods and simulation scenarios when the features are continuous and correlated.

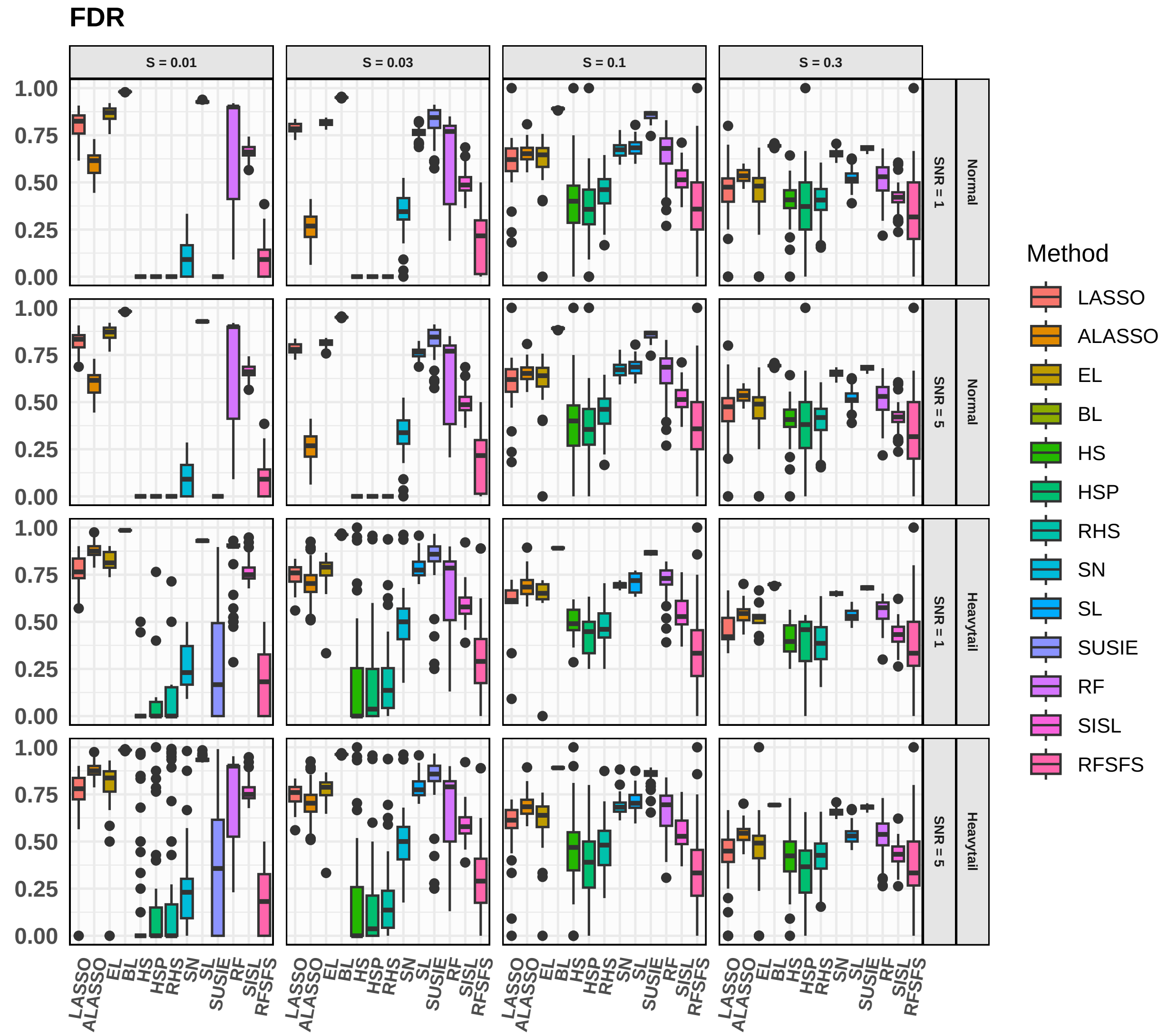

Figure S-5: The plot of FDR under different methods and simulation scenarios when the features are continuous and independent. $\sigma^2 = 1$

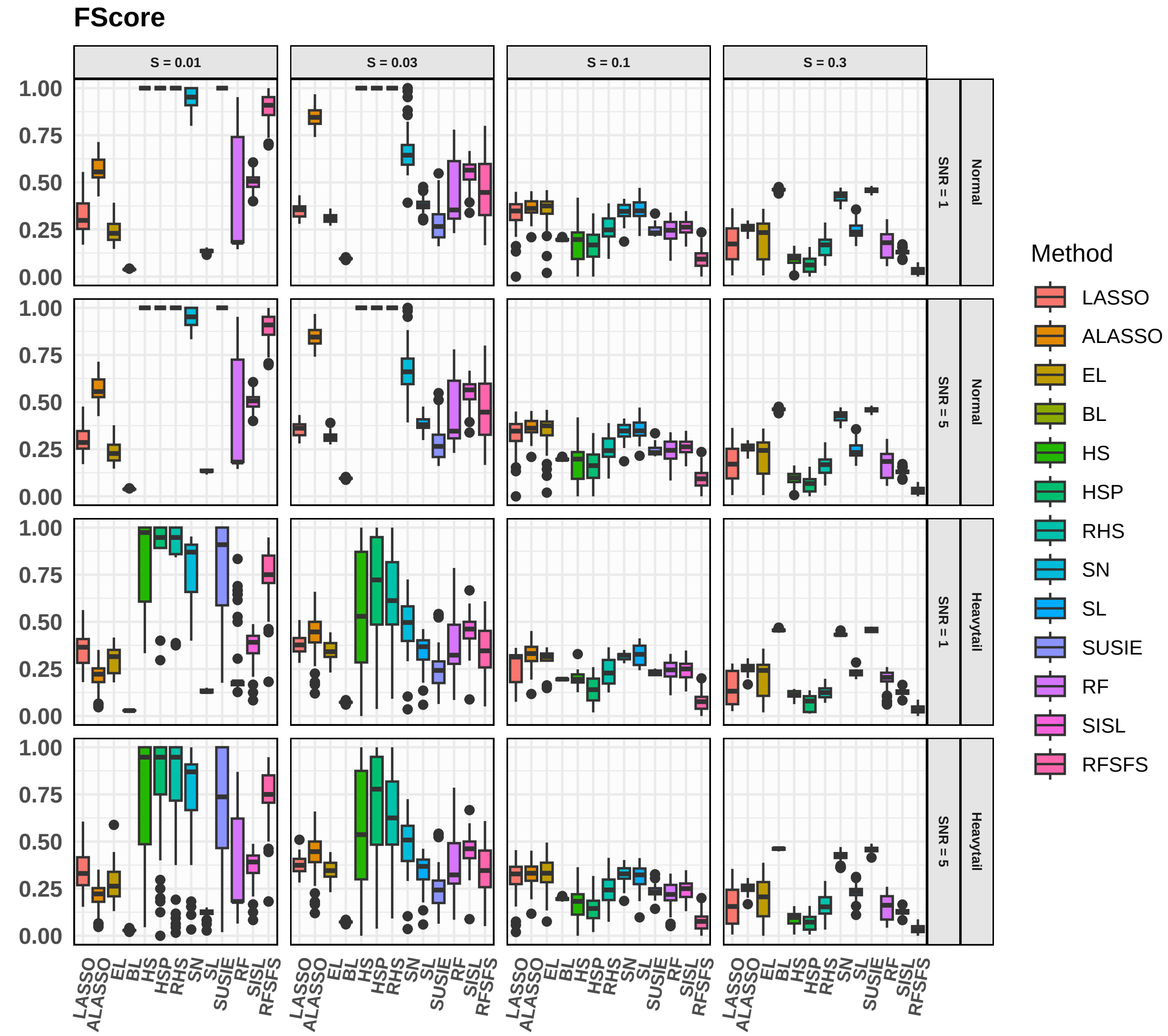

Figure S-6: The plot of Fscore under different methods and simulation scenarios when the features are continuous and independent. $\sigma^2 = 1$

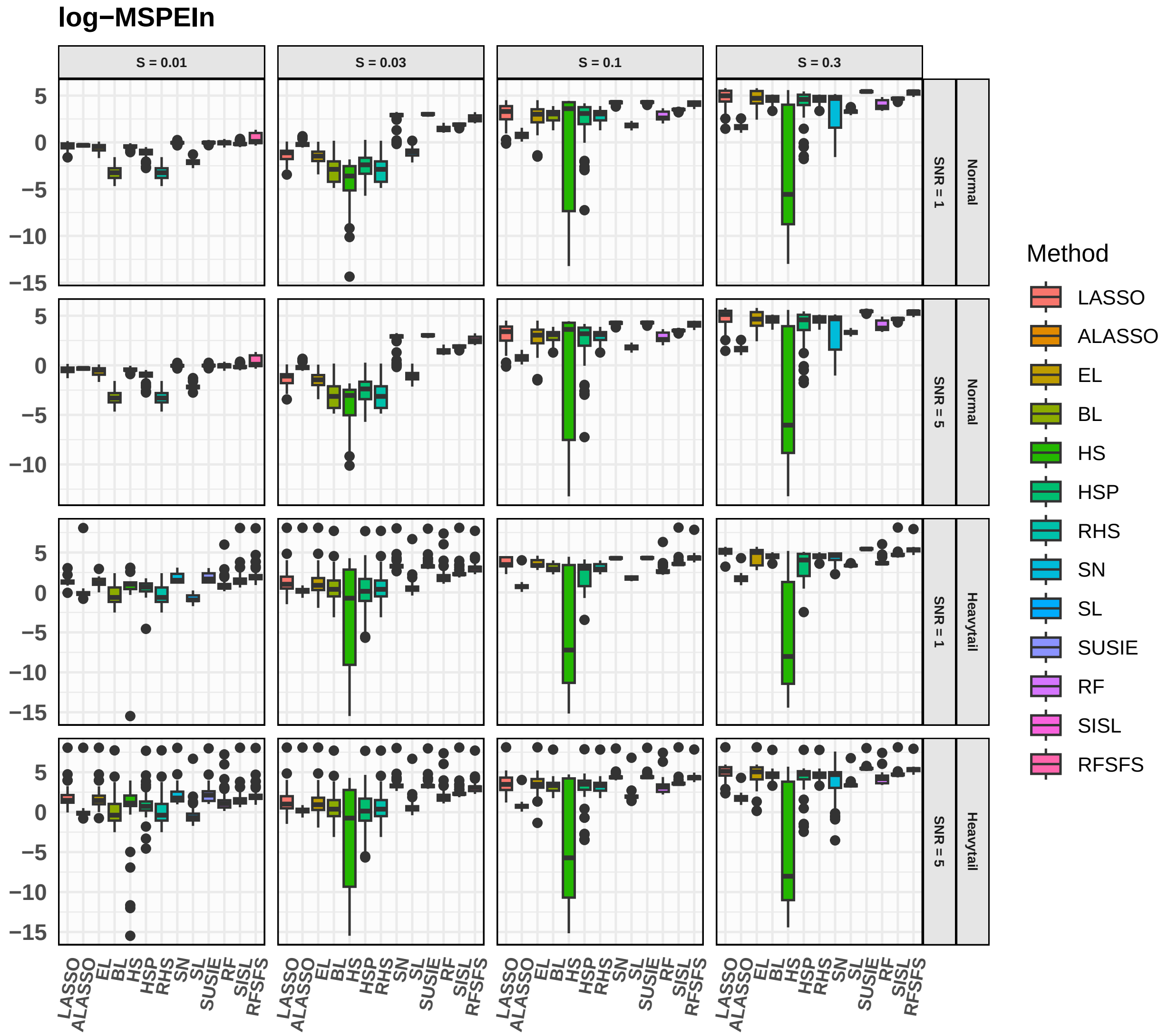

Figure S-7: The plot of MSPEIn under different methods and simulation scenarios when the features are continuous and independent. $\sigma^2 = 1$

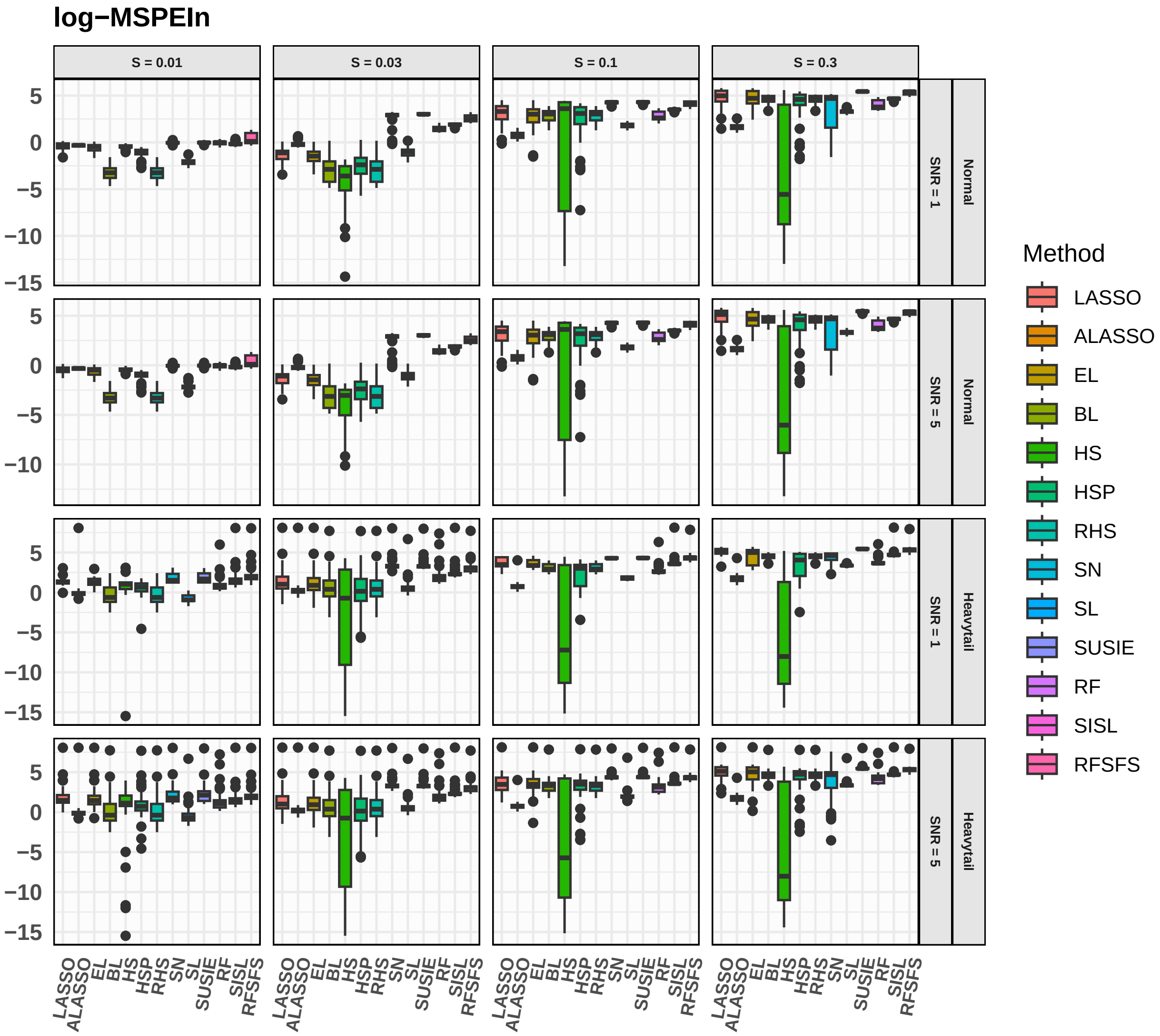

Figure S-8: The plot of MSPEout under different methods and simulation scenarios when the features are continuous and independent. $\sigma^2 = 1$

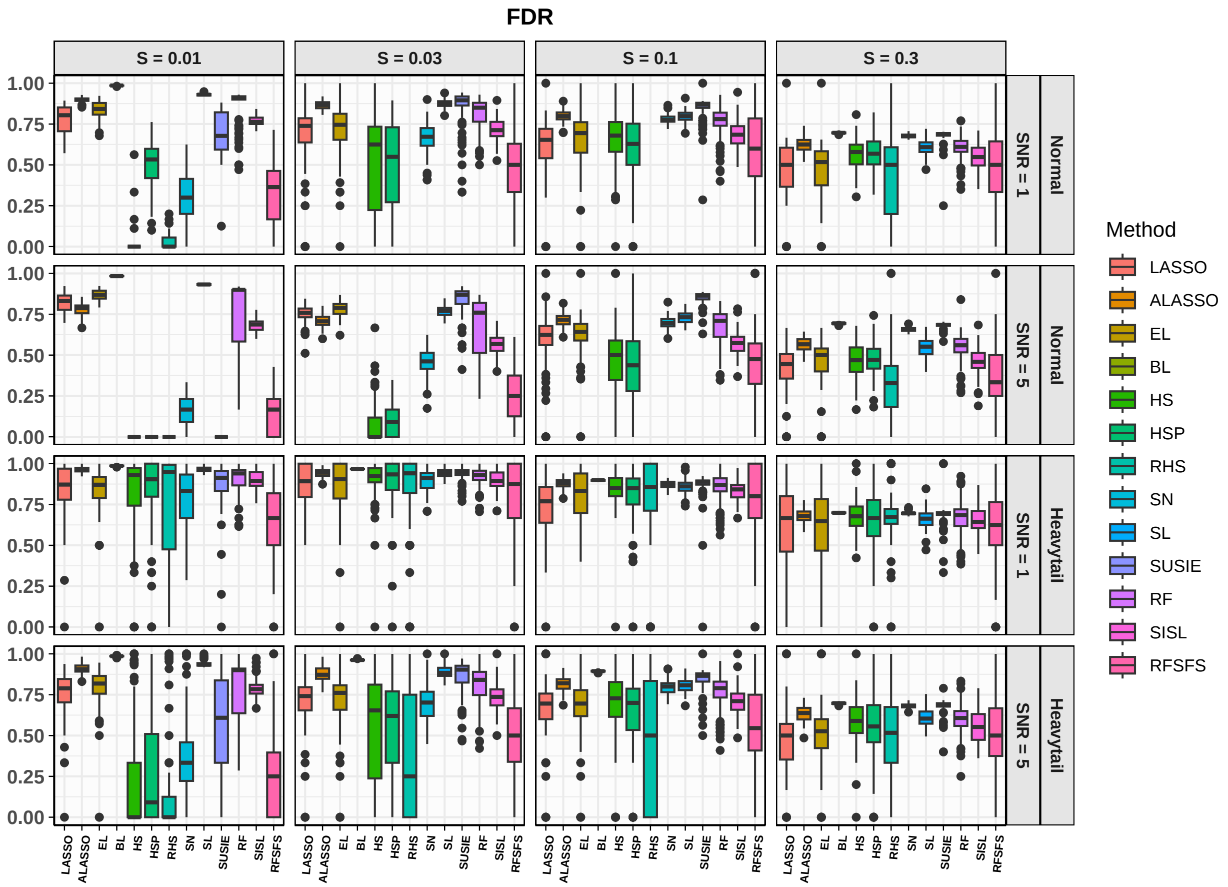

Figure S-9: The plot of FDR under different methods and simulation scenarios when the features are continuous and independent.

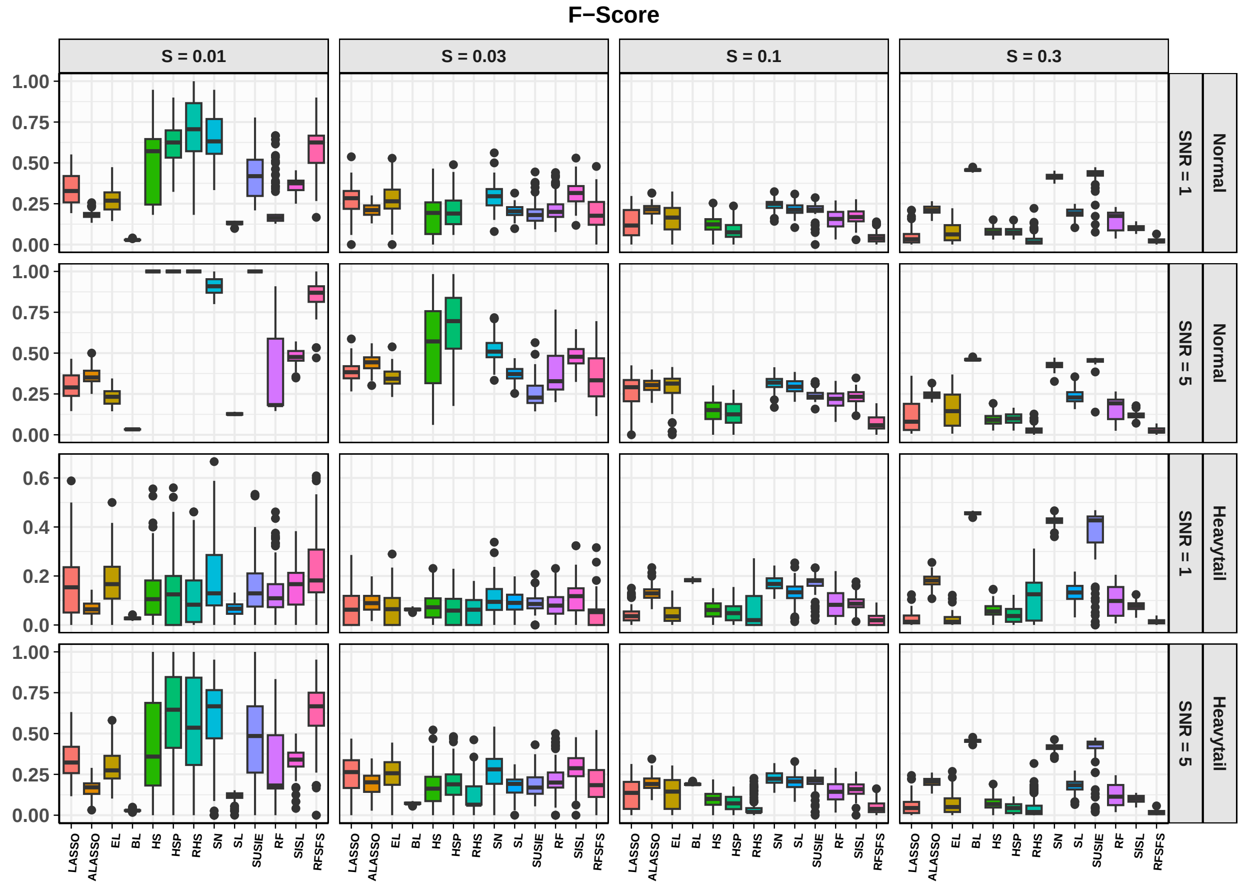

Figure S-10: The plot of Fscore under different methods and simulation scenarios when the features are continuous and independent.

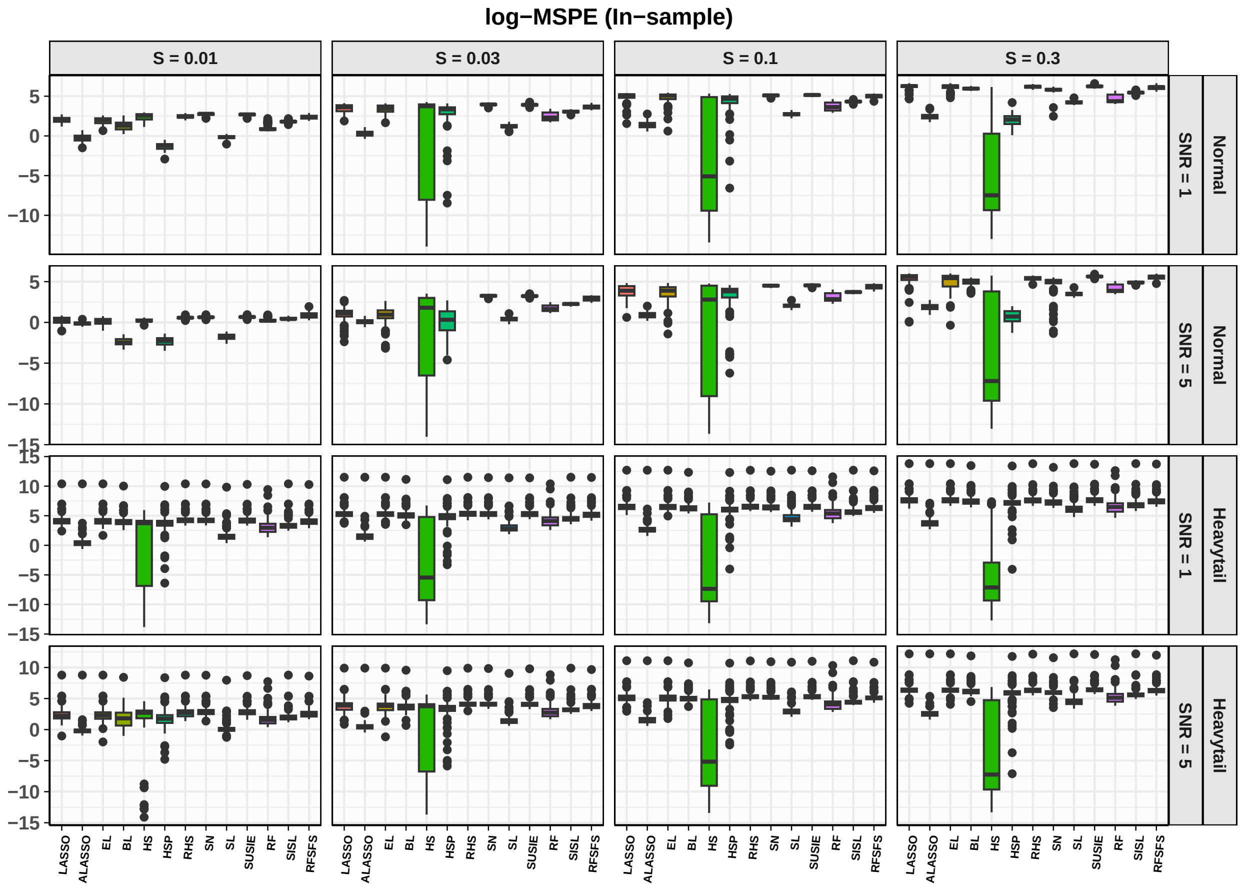

Figure S-11: The plot of MSPEIn under different methods and simulation scenarios when the features are continuous and independent.

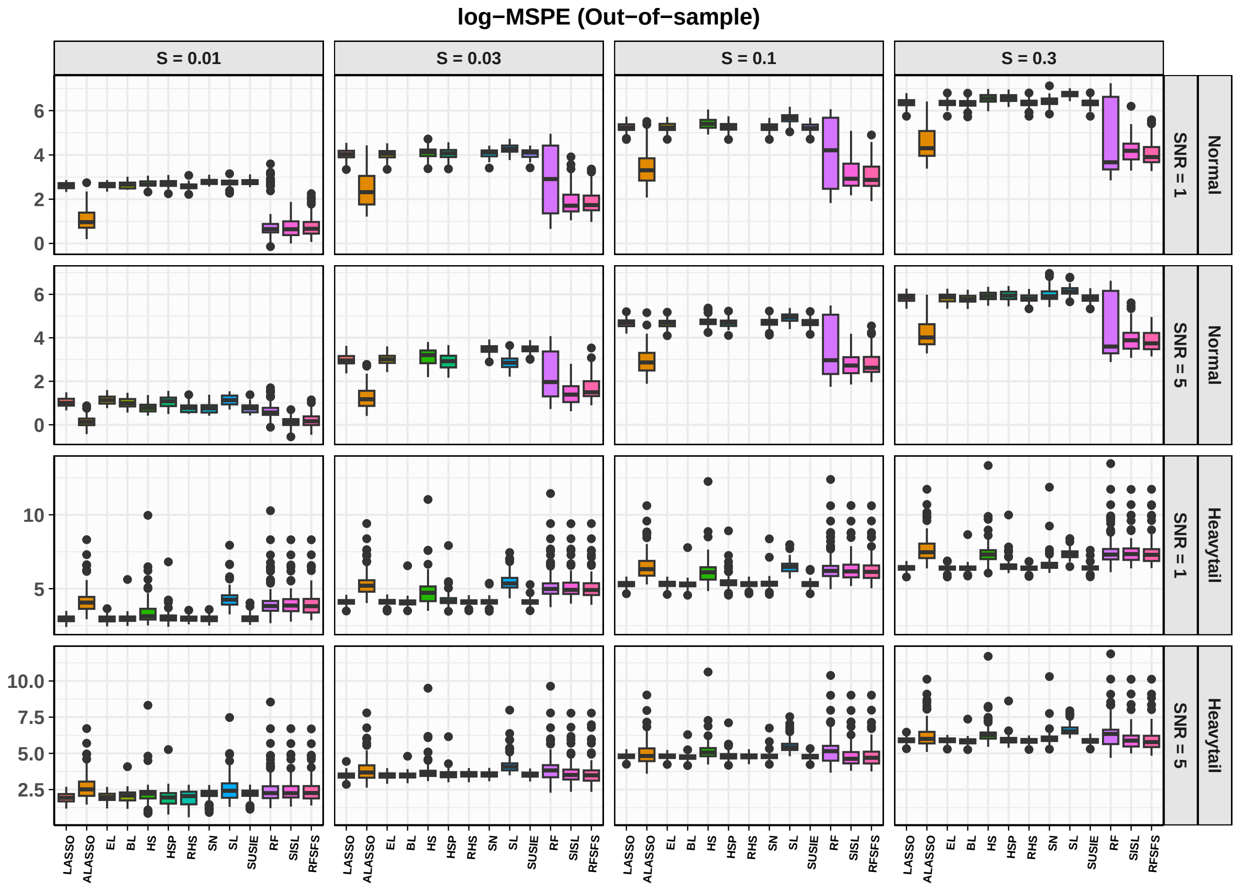

Figure S-12: The plot of MSPEout under different methods and simulation scenarios when the features are continuous and independent.

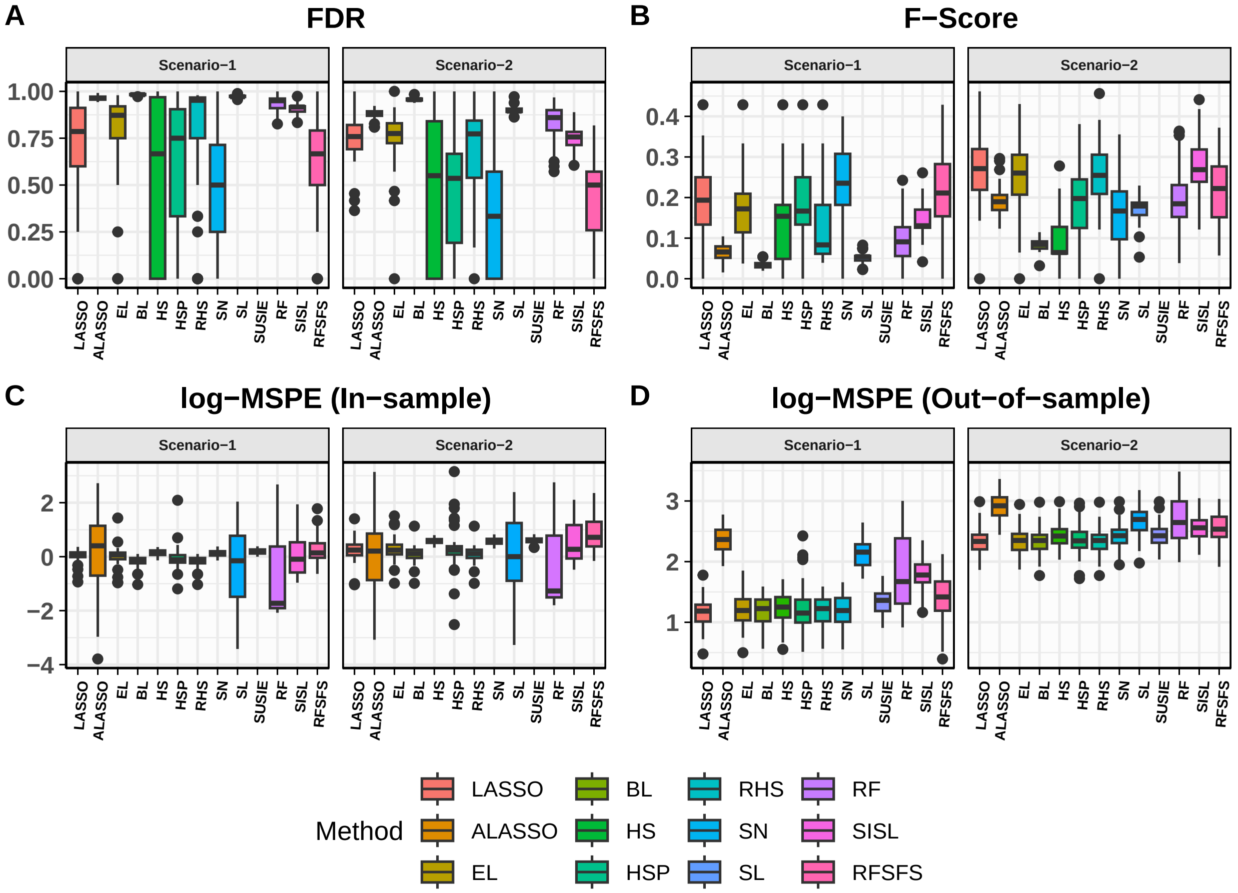

Figure S-13: The plot of FDR, Fscore, MSPEIn, MSPEout under different methods and simulation scenarios 1 and 2

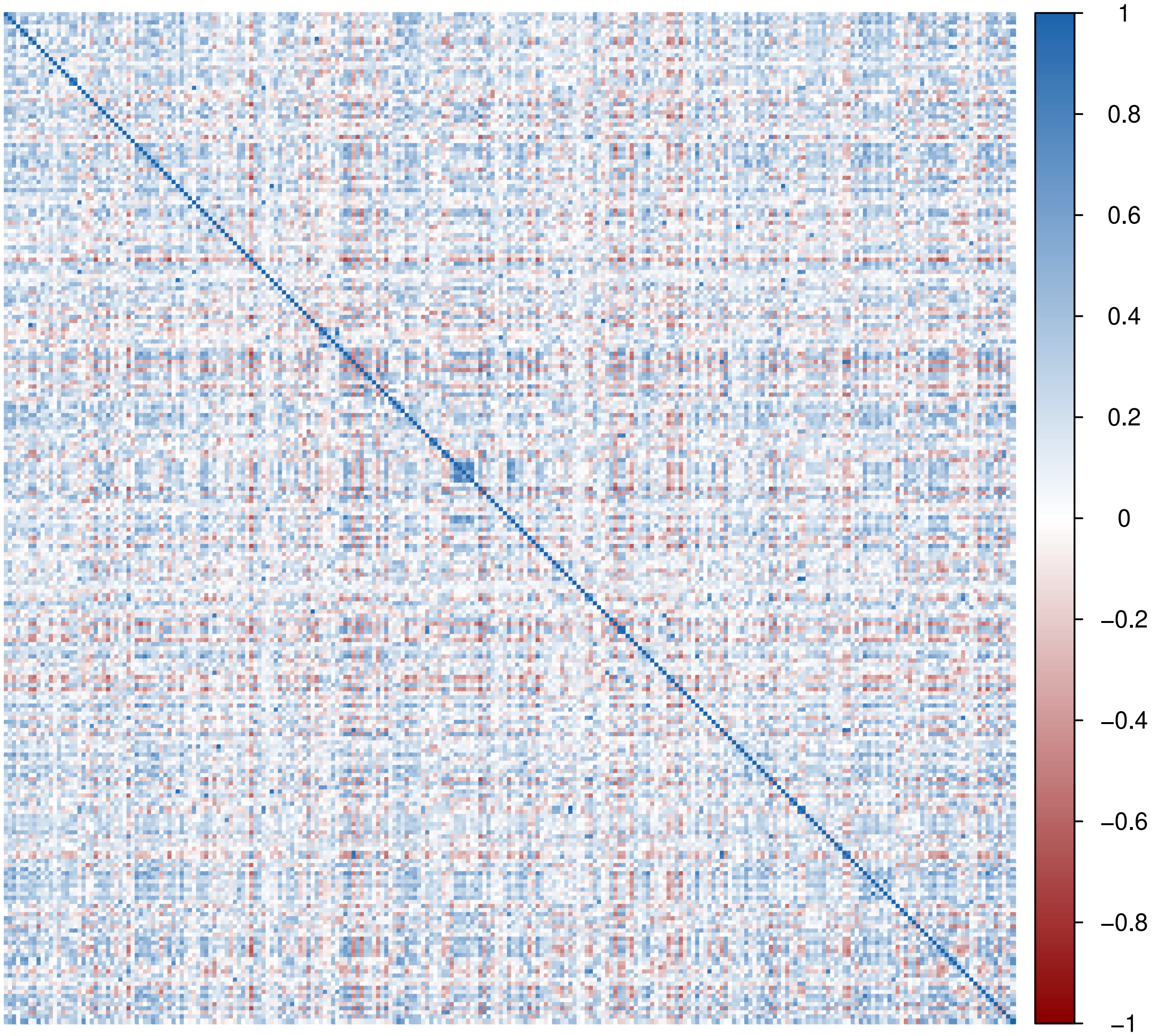

Figure S-14: The correlation plot of all 247 miRNAs included in the RCC data analysis.

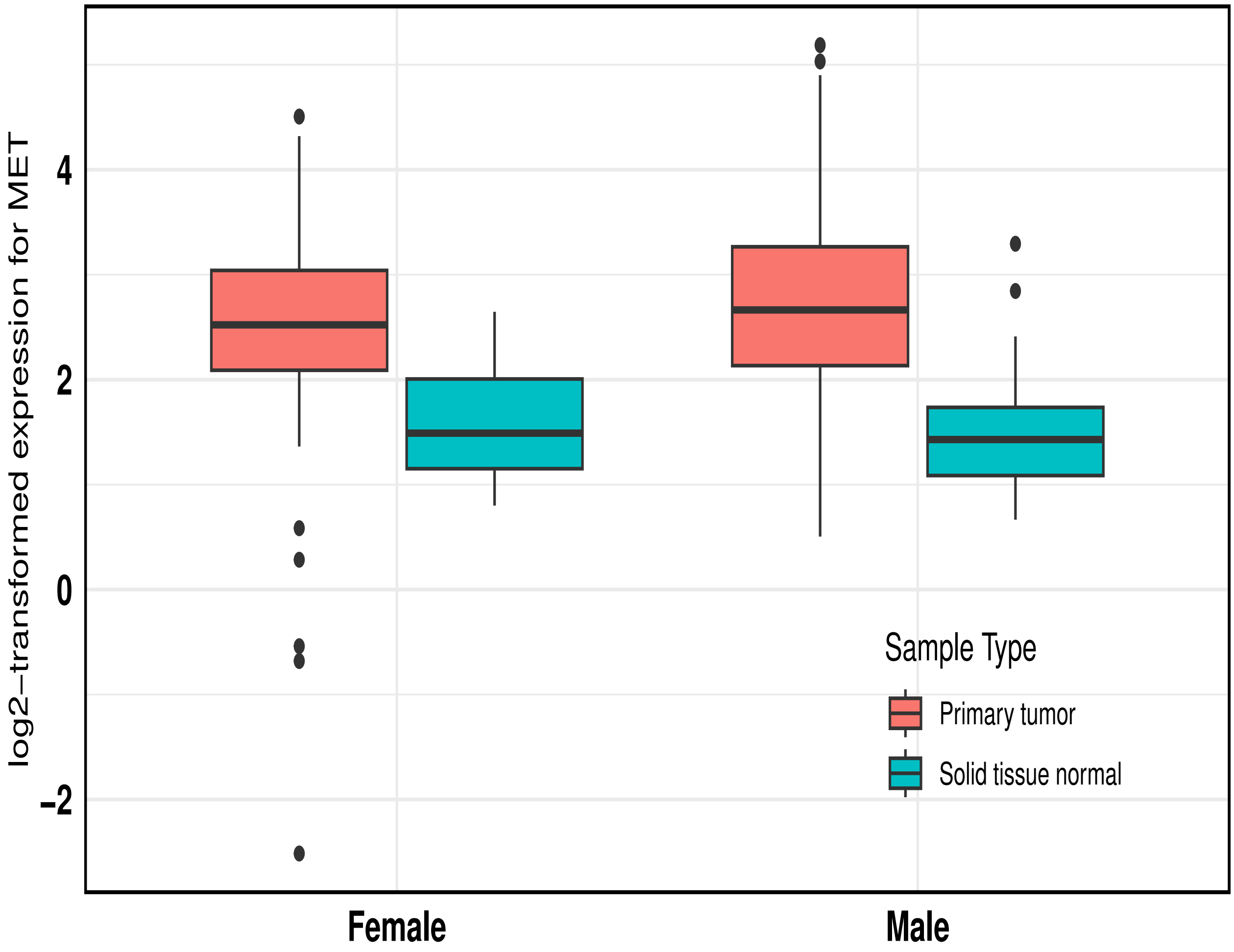

Figure S-15: Boxplots of the MET gene for both genders, primary tumor, and solid normal tissue.

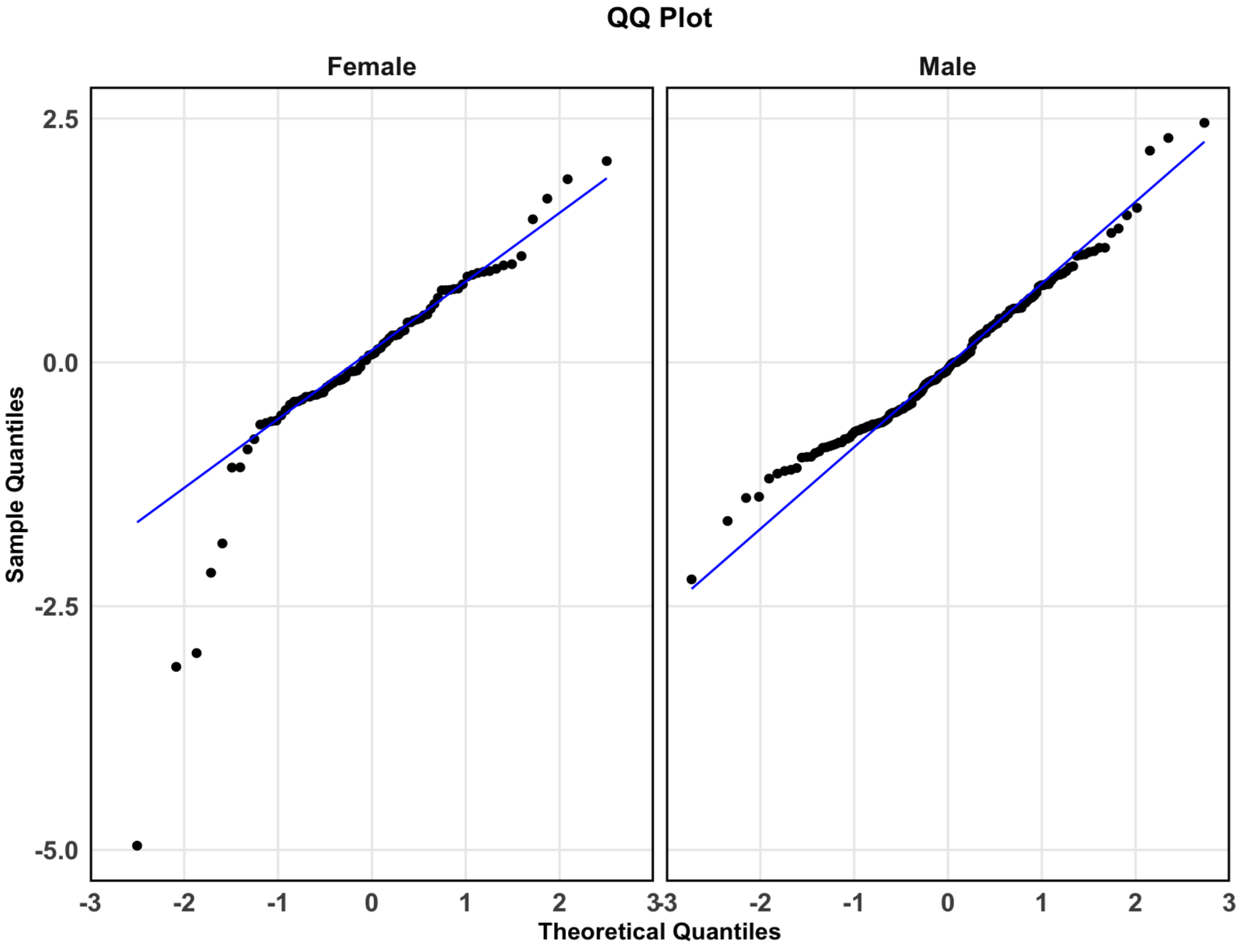

Figure S-16: QQplots of the MET gene for both genders.

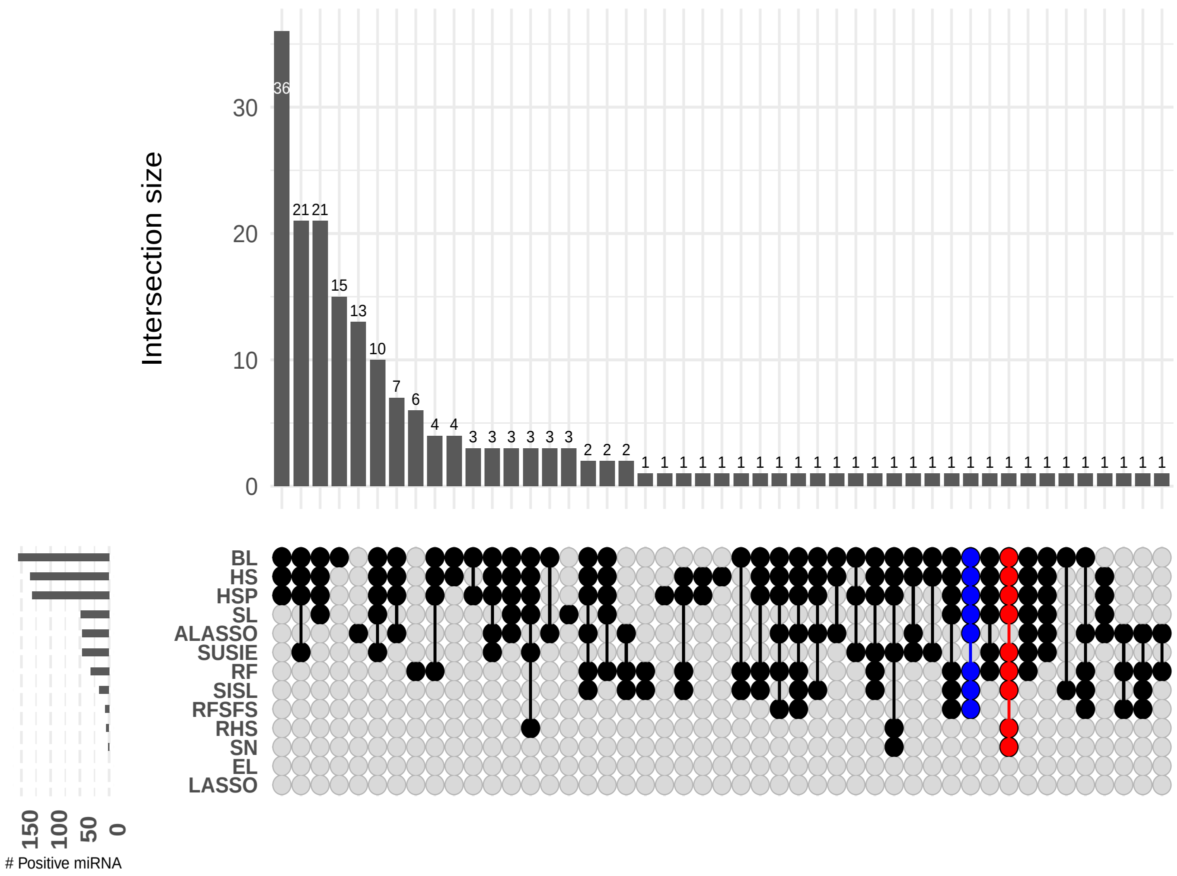

Figure S-17: The upset plot for the female group in the analyses of the RCC data.

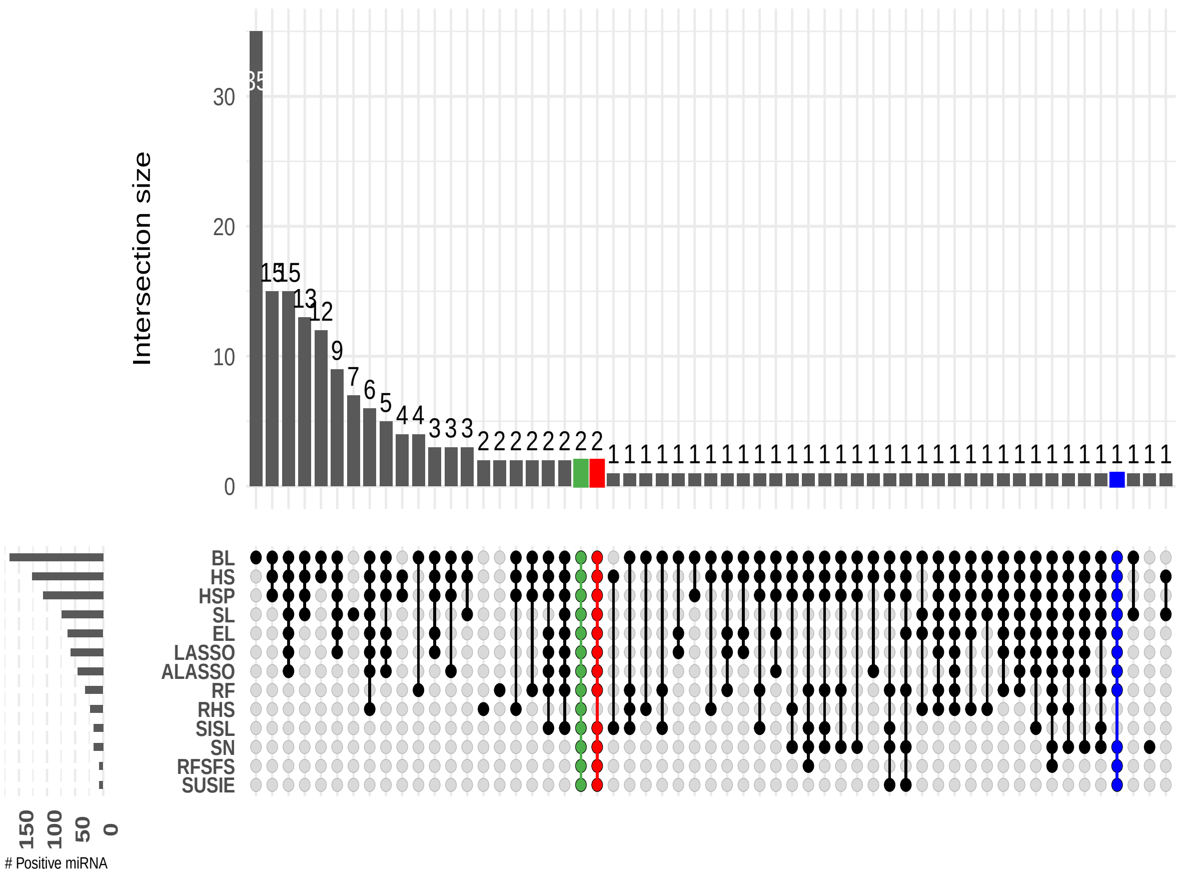

Figure S-18: The upset plot for the male group in the analyses of the RCC data.
